# Genetic admixture despite ecological segregation in a North African sparrow hybrid zone (Aves, Passeriformes, *Passer domesticus* × *Passer hispaniolensis*)

**DOI:** 10.1002/ece3.5744

**Published:** 2019-10-28

**Authors:** Martin Päckert, Abdelkrim Ait Belkacem, Hannes Wolfgramm, Oliver Gast, David Canal, Gabriele Giacalone, Mario Lo Valvo, Melita Vamberger, Michael Wink, Jochen Martens, Heiko Stuckas

**Affiliations:** ^1^ Senckenberg Naturhistorische Sammlungen Dresden, Senckenberg|Leibniz Institution for Biodiversity and Earth System Research Dresden Germany; ^2^ Laboratoire d'Exploration et de Valorisation des Écosystèmes Steppiques Faculté des Sciences de la nature et de la vie Université de Djelfa Djelfa Algeria; ^3^ Institute of Vertebrate Biology Brno & Masaryk University Brno Brno Czech Republic; ^4^ Department of Evolutionary Ecology Estación Biológica de Doñana—CSIC Seville Spain; ^5^ Centro para el Estudio y Conservación de las Aves Rapaces en Argentina (CECARA‐UNLPam) & Instituto de las Ciencias de la Tierra y Ambientales de La Pampa (INCITAP) Consejo Nacional de Investigaciones Científicas y Técnicas (CONICET) Santa Rosa Argentina; ^6^ Cooperativa Silene Palermo Italy; ^7^ Dipartimento di Scienze e Tecnologie Biologiche, Chimiche e Farmaceutiche Università degli Studi di Palermo Palermo Italy; ^8^ Department of Biology Institute of Pharmacy and Molecular Biotechnology Heidelberg University Heidelberg Germany; ^9^ Institute of Organismic and Molecular Evolution Johannes Gutenberg University Mainz Germany

**Keywords:** hybridization, introgression, microsatellites, mitochondrial DNA, Z‐chromosome

## Abstract

Under different environmental conditions, hybridization between the same species might result in different patterns of genetic admixture. Particularly, species pairs with large distribution ranges and long evolutionary history may have experienced several independent hybridization events over time in different zones of overlap. In birds, the diverse hybrid populations of the house sparrow (*Passer domesticus*) and the Spanish sparrow (*Passer hispaniolensis*) provide a striking example. Throughout their range of sympatry, these two species do not regularly interbreed; however, a stabilized hybrid form (*Passer italiae*) exists on the Italian Peninsula and on several Mediterranean islands. The spatial distribution pattern on the Eurasian continent strongly contrasts the situation in North Africa, where house sparrows and Spanish sparrows occur in close vicinity of phenotypically intermediate populations across a broad mosaic hybrid zone. In this study, we investigate patterns of divergence and admixture among the two parental species, stabilized and nonstabilized hybrid populations in Italy and Algeria based on a mitochondrial marker, a sex chromosomal marker, and 12 microsatellite loci. In Algeria, despite strong spatial and temporal separation of urban early‐breeding house sparrows and hybrids and rural late‐breeding Spanish sparrows, we found strong genetic admixture of mitochondrial and nuclear markers across all study populations and phenotypes. That pattern of admixture in the North African hybrid zone is strikingly different from i) the Iberian area of sympatry where we observed only weak asymmetrical introgression of Spanish sparrow nuclear alleles into local house sparrow populations and ii) the very homogenous Italian sparrow population where the mitogenome of one parent (*P. domesticus*) and the Z‐chromosomal marker of the other parent (*P. hispaniolensis*) are fixed. The North African sparrow hybrids provide a further example of enhanced hybridization along with recent urbanization and anthropogenic land‐use changes in a mosaic landscape.

## Graphical abstract

In this study, we investigate patterns of gene flow between the house sparrow, the Spanish sparrow, and their phenotypical hybrids on two study sites in Algeria. Unlike in European areas of sympatry, nuclear markers suggest strong genetic admixture across Algerian populations and across phenotypes.

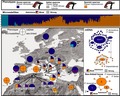

## 1 INTRODUCTION

Hybridization has recently become widely accepted as a driving force of speciation in animals and plants. Hybridization may increase genetic and/or phenotypic variability within short evolutionary timescales, leading to superior adaptation of hybrids in different contexts such as novel ecological niches (Schumer, Cui, Rosenthal, & Andolfatto, 2015). While this process may primarily result in a hybrid swarm (Seehausen, 2004), reproductive isolation is the crucial factor for the evolution of so‐called stabilized hybrid taxa. This can involve premating barriers, such as ecological niche preferences and behavior, and postmating barriers, such as genetic incompatibilities (Abbott et al., 2013; Arnold, 2016; Nolte & Tautz, 2010).

Hybridization can, however, drive speciation without resulting in hybrid species, that is, leading to reproductive isolation between evolutionary lineages. As long as reproductive barriers are incomplete, differently structured and often narrow hybrid zones can arise. In Europe, characteristic zoogeographic patterns of parapatry and secondary overlap have arisen from lineage separation in glacial refuges and postglacial range expansion (Hewitt, 2000, 2004; Schmitt, 2007). In these hybrid zones, interspecific gene flow is mediated by a complex interplay of different factors such as the strength of reproductive barriers or the timescale of contact. This interplay of factors can cause different hybridization outcomes such as reinforcement of reproductive barriers (Sevedio, 2004; Spencer, McArdle, & Lambert, 1986), hybrid swarm formation (Seehausen, 2004), or the emergence of hybrid species (e.g., fishes: Stemshorn, Reed, Nolte, & Tautz, 2011; birds: Barrera‐Guzmán, Aleixo, Shawkey, & Weir, 2018; Brelsford, Milá, & Irwin, 2011; Elgvin et al., 2011; Hennache, Rasmussen, Lucchini, Rimondi, & Randi, 2003; Hermansen et al., 2011; Lavretsky, Engilis, Eadie, & Peters, 2015; and mammals: Larsen, Marchán‐Rivadeneira, & Baker, 2014). Species with large distribution ranges and long evolutionary history may have experienced several secondary contact events in time, for example, via repeated recolonization from glacial refuges (Hewitt, 2000), and space, for example, via spatially separated hybrid zones across the distribution range (Aliabadian, Roselaar, Nijman, Sluys, & Vences, 2005). If the outcome of such independent hybridization events is not identical, different patterns of neutral and/or adaptive introgression might be observed in different regions of a species range (Barton & Hewitt, 1989; Curry, 2015).

**Figure 1:**
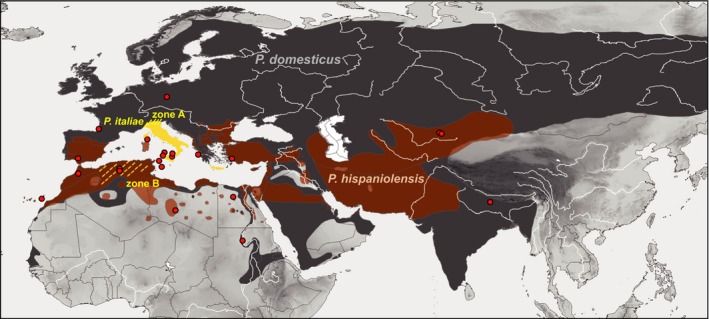
Palearctic distribution of the house sparrow (*Passer domesticus*: allopatric = gray shape), the Spanish sparrow (*Passer hispaniolensis*: light brown shape = allopatric; dark brown shape = sympatric with the house sparrow), and hybrid populations (yellow): stabilized hybrid form Italian sparrow (*Passer italiae*, on the Italian Peninsula, Corsica, Sicily, Malta, Crete, and other Mediterranean islands; zone A: hybrid zone with the house sparrow); mixed populations of phenotypic hybrids and the two parental species in North Africa (predominantly Algeria, zone B); red dots: sampled populations for genetic analysis

Early twenty‐first‐century studies have suggested that homoploid hybridization—without change in chromosome number—might be more frequent than previously believed and that it might act as an important driving force of evolution in animals (Abbott et al., 2013; Buerkle, Morris, Asmussen, & Rieseberg, 2000; Lamichhaney et al., 2017; Mallet, 2007; Mavárez & Linares, 2008; Schumer, Rosenthal, & Andolfatto, 2014). One well‐studied example is the Italian sparrow (*Passer italiae*), a stabilized homoploid hybrid form that resulted from interbreeding of the house sparrow (*Passer domesticus*) and the Spanish sparrow (*Passer hispaniolensis*) (Elgvin et al., 2011; Hermansen et al., 2011). The two parental species and their Italian hybrid are known to live in sympatry and allopatry (Figure 1) and are known to have experienced different evolutionary and demographic histories at postglacial and recent anthropogenic timescales (Ravinet et al., 2018; Sætre et al., 2012). On the Italian Peninsula, the Italian sparrow occupies a wide distribution range in the absence of either of the two parental species (Figure 1). In the Alps, a narrow contact zone exists (Figure 1: zone A) that is characterized by a steep cline of male plumage traits and shallower genetic cline between the Italian hybrid form and the house sparrow (Hermansen et al., 2011). In contrast, local contact between the Italian sparrow and its second parent is limited to a small introduced population of Spanish sparrows on Gargano Peninsula, where gene flow between the two taxa is restricted by strong ecological segregation and divergence of behavioral traits (Sætre et al., 2017). Previous field studies and genomic analyses show a scenario with a clear‐cut zoogeographic pattern of sympatry and parapatry of the two parental species and the Italian hybrid on the Eurasian continent that was shaped by complex interaction of (a) parental genetic incompatibilities (Elgvin et al., 2017; Eroukhmanoff et al., 2017; Hermansen et al., 2014; Trier, Hermansen, Sætre, & Bailey, 2014); (b) sexual selection on phenotypic traits (Bailey, Tesaker, Trier, & Sætre, 2015; Runemark, Fernández, Eroukhmanoff, & Sætre, 2018); and (c) selection on functional traits associated with ecological preferences (such as beak size and shape; Eroukhmanoff, Hermansen, Bailey, Sæther, & Sætre, 2013).

The situation in Eurasia strongly contrasts with the spatial mosaic of house sparrows, Spanish sparrows, and phenotypic hybrid populations in North Africa (Figure 1: zone B). That area has been subject to intense anthropogenic changes in past centuries, when intensification of farming and urbanization during past centuries has resulted in an eastward range expansion of the human commensal species, the house sparrow, allowing the coexistence with local Spanish sparrows and hence hybridization (Summers‐Smith & Vernon, 1972). The North African landscape is characterized by a dense mosaic of agricultural landscapes and human settlements separated by diverse types of steppe and desert (Hirche, Salamani, Abdellaoui, Benhouhou, & Martínez‐Valderrama, 2010). In this mosaic hybrid zone, the two sparrow species and their hybrids occupy suitable habitats in patchy anthropogenic landscape (such as crop fields, palm oases, villages, and cities) but avoid the interspersed inhospitable arid regions such as steppe and desert (Belkacem et al., 2016; Johnston, 1969). This spatial scenario is a singular phenomenon, because nowhere else both parental species and their phenotypic hybrids occur in close vicinity.

For the Algerian study populations of sparrows, recent studies revealed species‐specific differences in habitat and nesting site preferences and breeding phenology (Belkacem et al., 2016). Moreover, the mosaic hybrid zone is characterized by asymmetric introgression of the house sparrow mitogenome into phenotypic hybrids and even into Spanish sparrow populations on the adjacent crop fields (Belkacem et al., 2016).

To date, any assessment of genetic admixture based on nuclear markers is missing for the North African sparrow populations. In this study, we fill this gap using one mitochondrial marker, 12 microsatellite loci, and one z‐chromosomal marker (*CHD1Z*) to analyze patterns of genetic admixture in the North African hybrid zone. Based on the current state of knowledge outlined above, we predict that,

1. Phenotypic hybrids in Algerian mixed populations should be identifiable as genetic hybrids. Furthermore, these should show similar variation of the three genetic marker systems as the Italian hybrid form (e.g., near fixation of the house sparrow mitogenome and the Spanish sparrow *CHD1Z* alleles; Belkacem et al., 2016; Elgvin et al., 2011; Hermansen et al., 2011; see also Materials and Methods below)
2. Local spatial and temporal separation among Algerian house sparrows and Spanish sparrows (Belkacem et al., 2016) should act as a premating barrier and prevent interspecific gene flow to some degree. We thus expect admixture of the two nuclear markers to be limited in the two parental species despite strong unidirectional mitochondrial introgression in Algeria (Belkacem et al., 2016).
3. Algerian hybrids should be genetically more similar to house sparrows due to increased backcrossing in mixed urban populations. Likewise, the signal of admixture should be weaker in rural populations due to the absence of hybrids (Belkacem et al., 2016).

## 2 MATERIALS AND METHODS

### 2.1 Sampling and study sites in Algeria

**Figure 2:**
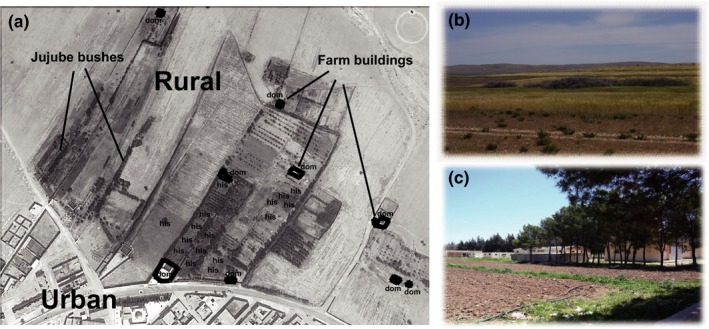
Map of study site at Hassi El Euch, Algeria; (a) aerial view on wheat fields in the north of the village modified from Google Earth; house sparrows (dom) occupy nesting sites in the urban area and in scattered farmhouses, whereas Spanish sparrows (his) breed in large colonies in jujube bushes; (b) agricultural site with stands of bushes; (c) Institut Technique Moyen Agricoles Spécialisés, study site at Djelfa

We sampled genetic material from 323 individuals from 21 collection sites across the trans‐Palearctic breeding ranges of our sparrow target species (including 16 populations with local samplings of *n* ≥ 5; Figure 1). Information on sample origin can be inferred from a material table provided at Dryad under https://doi.org/10.5061/dryad.v9s4mw6qf. The peculiar spatial scenario in the Algerian study populations is illustrated in Figure 2: At Hassi El Euch, an urban mixed population of house sparrows and phenotypic hybrids closely adjoins the rural area covered by large wheat fields in the north of the city (Figure 2b). In the crop fields, Spanish sparrows occupy stands of jujube bushes (*Zyziphus lotus*) in large breeding colonies, whereas only a few detached farmhouses provide nesting sites for house sparrows and hybrids (Figure 2a). At the study site in Djelfa, only house sparrows and hybrids occupy breeding sites in walls of buildings, for example, of the Institut Technique Moyen Agricoles Spécialisés (Figure 2c).

### 2.2 Phenotypic classification

**Figure 3:**
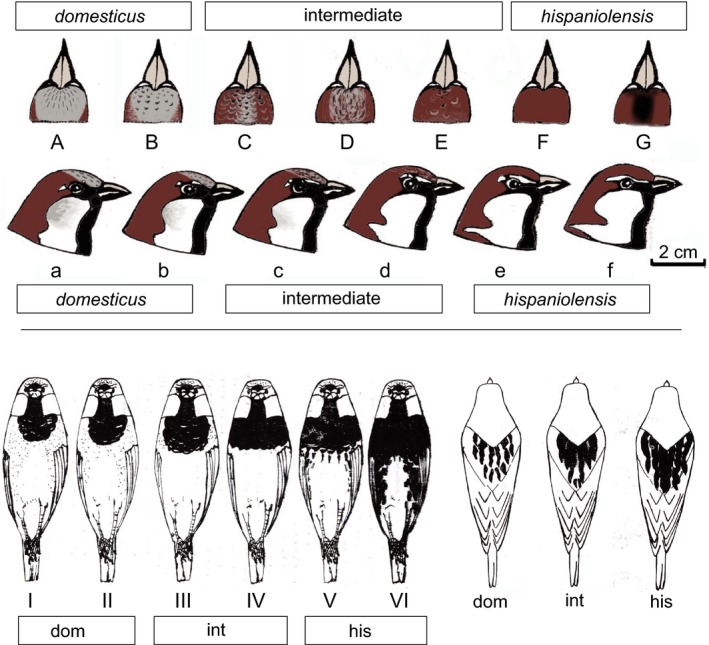
Phenotypic variation of North African house sparrows, Spanish sparrows, and hybrids, rearranged from original drawings by A.A. Belkacem; traits: dorsal view, A–G: crown and neck, 100% gray = *domesticus*, 0% gray = *hispaniolensis*; lateral view, a–f: cheek, 100% grayish = *domesticus*, 0% grayish = *hispaniolensis*; note also clinal variation of supercilium and collar stripe; ventral view, I–VI: black breast patch small central = *domesticus*, large across entire breast = *hispaniolensis*; flanks, entirely without stripes = *domesticus*, intensely striped = *hispaniolensis*; lower right: coloration of the back (phenotypes: *domesticus* [dom], *hispaniolensis* [his], *intermediate* [int])

In previous studies, phenotypic diagnosis of the house sparrow, Spanish sparrow, and the Italian sparrow has mainly relied on male plumage color patterns mostly of the crown, the cheek, and the back (Bailey et al., 2015; Hermansen et al., 2011; Runemark, Bailey, Bache‐Mathiesen, & Sætre, 2018). Compared to Italian sparrow populations, Rothschild and Hartert (1912) described a great phenotypic diversity of North African sparrow populations and distinguished 20 different head color patterns. For this study, we based individual classification of phenotypes on six phenotypic traits (crown, neck, cheek, breast, flanks, and back; as done by Belkacem et al., 2016). We distinguished (a) seven different crown color patterns and six different facial color patterns, and (b) six different ventral and three different dorsal plumage patterns (Figure 3). Only males that showed the typical species‐specific color pattern for all six traits were classified as either of the two parental species (as applied in Belkacem et al., 2016). Based on the different combination of plumage traits, we calculated an individual hybrid score from 0 (*P. domesticus*) to 1 (*P. hispaniolensis*), for example, a male with five traits showing the typical house sparrow phenotype and one trait being intermediate scored 0.0833 (Table S1). Based on the same system, we classified Italian sparrows using photographs available from fieldwork (Figure S1).

Hybrid scores were calculated and compared for males only, because unanimous assignment of females to either of the parental species is critical and highly prone to error (Elgvin et al., 2011; Hermansen et al., 2011; biometric analysis in Belkacem et al., 2016). In accordance with Hermansen et al. (2011), the few females that were included in the Algerian sampling were only assigned to the urban or rural study populations but not to a particular phenotype.

### 2.3 Choice of molecular markers

To study patterns of admixture in the North African hybrid zone, we chose three different marker systems. First evidence of the hybrid origin of the Italian sparrow was inferred from one mitochondrial marker (NADH dehydrogenase subunit 2; *ND2*) and microsatellites (Hermansen et al., 2011). The set of microsatellites used here (Table S2) has been developed specifically for sparrows and has been proven to (a) reliably distinguish the parental species (*P. domesticus* and *P. hispaniolensis*) from each other and from the admixed Italian hybrid form (Hermansen et al., 2011) and (b) reveal intraspecific diversification in Australian house sparrows (Sheldon, Schrey, Andrew, Ragdsale, & Griffith, 2018). For the mitochondrial marker *ND2*, there has been firm evidence of strongly asymmetrical introgression of the house sparrow mitogenome into both the Italian sparrow (Hermansen et al., 2011) and North African sparrow hybrids (Belkacem et al., 2016). As a third marker, we chose the sex chromosomal *CHD1Z*, a nuclear locus that seems to be under positive, divergent selection in the two parental sparrow species (Elgvin et al., 2011). In genetic cline analyses, *CHD1Z* and four other Z‐linked loci exhibited restricted introgression in parental sympatry compared to autosomal markers. These Z‐linked genes were thus suggested as candidate genes potentially associated with reproductive isolation (Hermansen et al., 2014; Trier et al., 2014).

Overall, our set of markers allows for a clear distinction of the Italian hybrid form from its two parental species: In the Italian sparrow, the mtDNA of one parent (*P. domesticus*) and the Z‐chromosomal marker of the other parent (*P. hispaniolensis*) are fixed (Elgvin et al., 2011; Hermansen et al., 2011). We therefore consider the selected markers a strong combination for the study of sparrow hybrid populations in North Africa. Though house sparrow mtDNA is not entirely fixed in North African hybrids (Belkacem et al., 2016), we expect these hybrid populations to have a similar genetic constitution like Italian hybrids, that is, show strong admixture of microsatellite loci and near fixation of z‐chromosomal alleles of the Spanish sparrow.

### 2.4 Wet‐lab analysis and Sanger sequencing

NADH dehydrogenase subunit 2 (*ND2*) sequences from 197 of our samples were already available to us from Belkacem et al. (2016). Here, we amplified and sequenced *ND2* for 126 additional samples, belonging to eleven additional populations, to complete the mitochondrial data set for our total sampling (323 samples from 16 populations). DNA extraction, standard primer combinations, PCR, and sequencing protocols followed Belkacem et al. (2016).

For identification of two putative heteroplasmic Italian sparrows, we amplified a 529 bp fragment of ND2 with specifically designed primer pairs: PassdomspecF = 5′‐GAG GTA TTG CAA GGT TCA CCT C‐3′, PassdomspecR = 5′‐GCA ACA ATT ACA CTG CCC CCT CAC‐3′; for the Spanish sparrow: PasshisspecF = GAA GTG CTG CAA GGT TCA CCC, PasshisspecR = CAC GAC AAT TAC ACT ACC CCC TCA/T3′ (for laboratory protocols and results, see Päckert, Giacaolone, Lo‐Valvo, & Kehlmaier, 2019).

We amplified *CHD1Z* from 297 samples with PCR primers CHD1Z‐F (5′‐TAG AGA GAT TGA GAA CTA CAG T‐3′) and CHD1Z‐R (5′‐GAC ATC CTG GCA GAG TAT CT‐3′; Borge, Webster, Andersson, & Sætre, 2005; Elgvin et al., 2011). One PCR volume of 25 μl contained 15.8 μl ddH2O, 3.0 μl buffer, each 1.0 μl forward and reverse primer (10 μM), 1.0 μl dNTP (10 mM), 3.0 μl template DNA, and 0.2 μl Taq DNA polymerase. The PCR profile was as follows: initial degradation for 30 s at 98°C, 35 cycles of 8 s at 98°C, 30‐s annealing at 53°C, and 30‐s elongation at 72°C, with a final elongation step for 30 s at 72°C. PCR products were visualized on agarose gels including GelRed™ (Biotium) and were purified using ExoSAP^®^ (Affymetrix^®^). Labeled PCR fragments were run on a 16‐column ABI 3130xl capillary sequencer (Applied Biosystems).

### 2.5 DNA sequence analysis

All sequences (*ND2* and *CHD1Z*) were aligned manually using MEGA 5.1 (Tamura et al., 2011). Variable and ambiguous sites were checked visually for accuracy and validated by examining the raw data electropherogram output file. For *CHD1Z* sequences with at least one heterozygous site, haplotypes were assigned statistically using the PHASE 2.1.1 algorithm (Stephens & Donnelly, 2003; Stephens, Smith, & Donnelly, 2001) implemented in DnaSP 5.10.01 (Librado & Rozas, 2009). Nucleotide sequences of the coding mtDNA marker (*ND2*) were translated into protein sequences with MEGA 5.1 in order to control for stop codons and, thus, to exclude numts (nuclear copies of mitochondrial genes) as a potential source of error. Minimum‐spanning networks of *ND2* haplotypes and *CHD1Z* alleles were reconstructed with TCS v. 1.2 with gaps treated as fifth state (Clement, Posada, & Crandall, 2000).

We also used Arlequin 3.5 to calculate diversity indices (such as haplotype and nucleotide diversity for *ND2* and observed and expected heterozygosities [*H*_O_, *H*_E_] for *CHD1Z*). To avoid drawbacks due to haplodiploidy of the Z‐chromosome (Hermansen et al., 2014), we excluded females from calculations of diversity indices of the z‐chromosomal marker *CHD1Z* (for which only males are diploid).

### 2.6 Microsatellite analysis

We designed a new multiplex microsatellite protocol for 13 microsatellite loci using published information about the microsatellite primers (Table S2). To minimize differences of annealing temperature (*T*_m_) and to maximize spacing between markers with overlapping fluorescence spectra (Guichoux et al., 2011), we divided 13 primer pairs into three multiplex sets A, B, and C (Table S2). For multiplex PCR, we used the Type‐it^®^ Microsatellite PCR Kit (Qiagen) following the manufacturer's instructions. To increase the number of possible reactions, the protocol was modified as follows: The reaction setup for each multiplex PCR had a total volume of 10 μl containing 5 μl Multiplex Master Mix (HotStar*Taq* DNA polymerase, 6 mM MgCl_2_, and dNTPs), 3 μl RNase‐free water, 1 μl of each respective primer mix, and 1 μl DNA template (<200 ng DNA/reaction).

A total of 323 samples were genotyped for 13 unlinked microsatellite loci. Locus Pdo27 had to be excluded from further analyses due to ambiguities during allele scoring; thus, 12 microsatellite loci remained to estimate population genetic parameters. Alleles were scored manually using GeneMapper 4.0 (Applied Biosystems) using a modified method proposed by Amos et al. (2007) and Guichoux et al. (2011). To visualize allele classes and identify problematic alleles, cumulative frequency plots of size distribution were constructed for all scored alleles of each microsatellite. To transform the data, we used Convert 2.0 software (Glaubitz, 2004).

We used Arlequin 3.5 (Excoffier & Lischer, 2010) to calculate linkage between alleles for the 16 sparrow populations with samplings of *n* > 5. We also used Arlequin 3.5 to calculate locus‐specific observed and expected heterozygosities (*H*_O_,* H*_E_) for each sample population, and pairwise *F*_ST_ (fixation index) values among populations and to test for departure from Hardy–Weinberg equilibrium (HWE) and for linkage disequilibrium (LD). We performed an exact test of HWE based on a Monte Carlo Markov chain (MCMC) length of 106 repetitions and 105 dememorization steps. To test whether loci were in LD, the number of permutations was set on 104. LD and HWE tests were adjusted with sequential Bonferroni correction to minimize the chance of a type one error (Rice, 1989). Although deviations from Hardy–Weinberg expectations and linkage equilibrium were found in single cases, none of the loci persistently showed departure from HWE and there was no significant linkage between pairs of loci throughout all populations (Table S3). Thus, we used all markers for population genetic analyses.

Nonspatial Bayesian inference of population structure was performed using the software STRUCTURE 2.3.3. (Falush, Stephens, & Pritchard, 2003; Pritchard, Stephens, & Donnelly, 2000). STRUCTURE runs were alternatively performed (a) under the admixture ancestry model and correlated allele frequencies and (b) under a LOCPRIOR model that allows for classification of the individuals into groups, which are given to the algorithm as an a priori parameter (Hubisz, Falush, Stephens, & Pritchard, 2009). The LOCPRIOR model was run under two different settings: (a) classifying individuals according to the phenotype (house sparrow, Spanish sparrow, or hybrid) and (b) using geographic origin as LOCPRIOR.

All STRUCTURE analyses were conducted for 1–10 putative genetic clusters (*K*) with ten runs for each value of *K*. We performed 10^6^ iterations per run with a burn‐in period of 5 × 10^5^ steps. For further processing of the STRUCTURE output, we used STRUCTURE HARVESTER (Earl & vonHoldt, 2012) and plotted the mean likelihood over 10 runs for each *K*. To select the most likely number of genetic clusters (*K*), we used the approach by Evanno, Regnaut, and Goudet (2005) based on the rate of change of the likelihood function with respect to *K*. STRUCTURE analysis was also used to estimate the extent of genetic admixture in different populations following the method described by Randi (2008). As a measure of admixture, we relied on mean assignment probabilities *q* (0 < 1) and 95% probability intervals (PI) of *q* inferred from STRUCTURE output (Figure S2). To test the resolution power of the Bayesian approach STRUCTURE for inferring hybrid status and pure ancestry, simulations were run using HYBRIDLAB 1.0 (Nielsen, Bach, & Kotlicki, 2006). We chose allopatric populations of parental species from regions beyond the range of sympatry as representatives of parental genotypes (house sparrow: 17 samples from Germany; Spanish sparrow: 22 samples from Spain). Using these data, 20 genotypes of each hybrid class (F1, F2, and the two backcrosses) were modeled in HYBRIDLAB 1.0. Then, the obtained simulated hybrid data were subjected to analyses using STRUCTURE, together with the data of the two parental populations from Germany and Spain (see Vamberger et al., 2017, for a similar approach).

## 3 RESULTS

### 3.1 Genetic admixture

**Figure 4:**
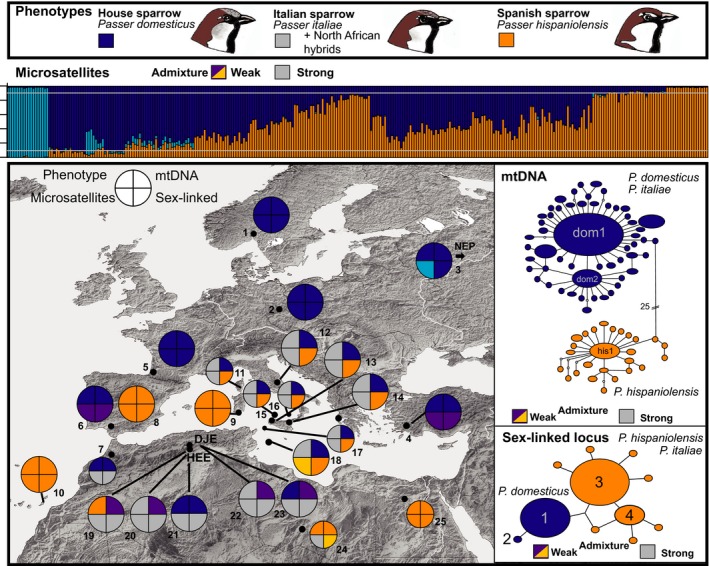
Phenotypic and genetic variation among study populations (including combined data by Hermansen et al., 2011 and Elgvin et al., 2011; populations 1, 9, and 12); house sparrow, *Passer domesticus*: (1) Norway, (2) East Germany, (3) Nepal, (4) Turkey, (5) France, (6) Spain, (7) Morocco, (21) Algeria, Hassi El Euch, and (23) Algeria, Djelfa; Spanish sparrow, *Passer hispaniolensis*: (8) Spain, (9) Sardinia, (10) Fuerteventura, (19) Algeria, Hassi El Euch, (24) Libya, and (25) Egypt; *Passer italiae*: (11) Corsica, (12) Italy mainland, (13) Fraginesi, (14) Maletto, (15) Ustica, (16) Lipari, (17) Pantelleria, and (18) Lampedusa; genetic markers: microsatellites = STRUCTURE plot for *k* = 3 under the LOCPRIOR model (populations) for the entire data set including the population from Nepal; weak admixture: for a mean assignment probability range of 0.1 < *q* < 0.3 for most individuals = close to *P. domesticus* (violet), for a range of 0.7 < *q* < 0.9 = close to *P. hispaniolensis* (yellow); mtDNA = *ND2*, haplotype network 674 bp; sex chromosomal marker = *CHD1Z*, haplotype network 385 bp

**Figure 5:**
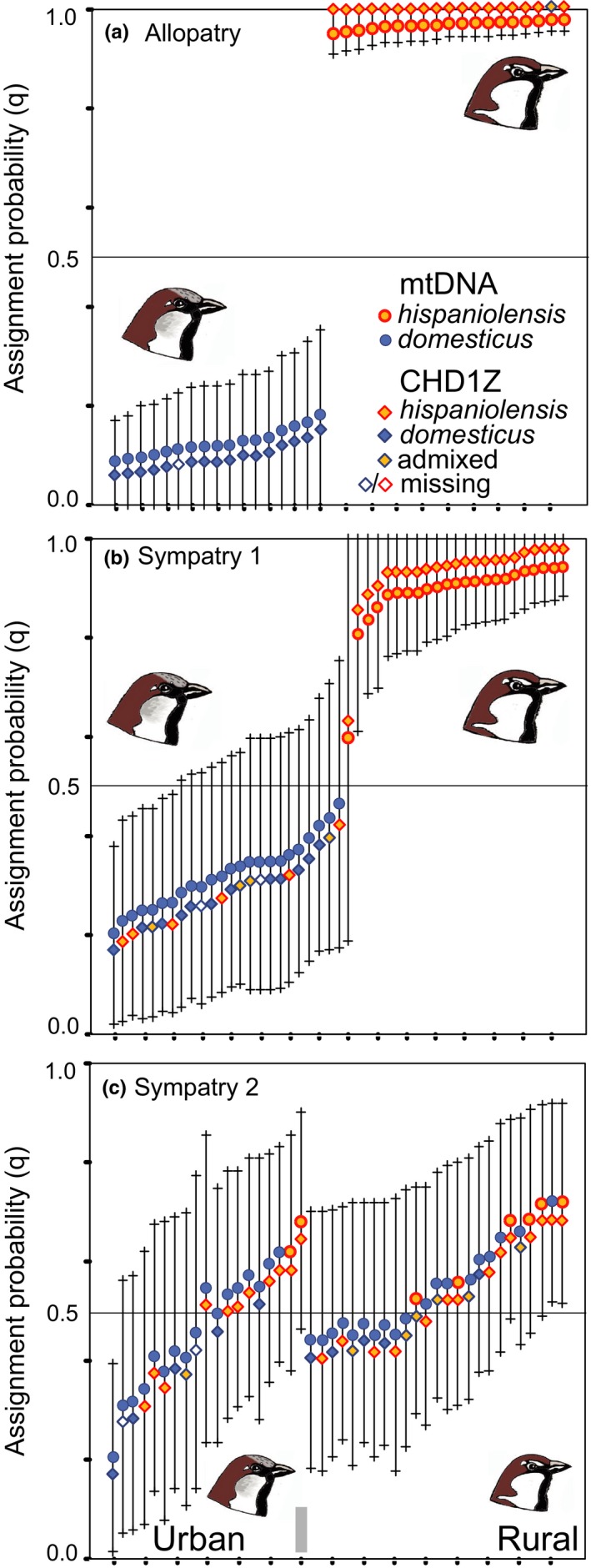
Divergence and admixture of house sparrows (*Passer domesticus*) and Spanish sparrows (*Passer hispaniolensis*) for three scenarios of (a) allopatry (Germany vs. Fuerteventura) and sympatry (b) on the Iberian Peninsula, (c) in North Africa, at study site Hassi El Euch; *y*‐axis: assignment probabilities from STRUCTURE analysis with 12 microsatellite loci; each line represents an individual's 95% PI for *q*‐values (13 microsatellite loci; assignment to the Spanish sparrow cluster relative to the house sparrow cluster; in ascending order from 0 = house sparrow ancestry to 1 = Spanish sparrow ancestry); *ND2* haplotypes of each individual indicated by colored dots; *CHD1Z* allele combinations for each individual indicated by colored diamonds: *hispaniolensis* = combinations of alleles 3–11; *domesticus* = combination allele 1/1; admixed = combination allele 1 and any of allele 3–11

Based on 12 microsatellite loci, Evanno's ∆*k* identified two clusters (*k* = 2) as the most plausible population structure when using the complete data set (*n* = 323) under the admixture–frequency‐correlated model. The house sparrow population from Nepal was separated from all other populations, whereas Western Palearctic house sparrows, Spanish sparrows, and Italian sparrows were not distinguished as separate clusters (plot not shown). However, the result for *k* = 3 from the same run yielded a biological meaningful scenario: Again, the Himalayan house sparrow population appeared as a separate cluster, but Western Palearctic populations of house sparrows (Germany, France) and Spanish sparrows (Fuerteventura, Egypt) were distinguished as a second and third cluster (Figure 4, STRUCTURE plot for *k* = 3). Most other populations showed a signal of genetic admixture. Based on the results above, we performed a second STRUCTURE analysis excluding the Nepal house sparrow population. Using this reduced Western Palearctic data set (*n* = 301) under the admixture–frequency‐correlated model, the two parental species were recovered as distinct genetic clusters with *k* = 2 as the most plausible population structure (Figure S3). Populations from the allopatric ranges of the two parental species did not show any sign of admixture (Figure S3) and 95% PIs of *q*‐values did not overlap (e.g., among German house sparrows and Canary Island Spanish sparrows: Figure 5a). HYBRIDLAB calculations indicated that sparrows with mean assignment probabilities of *q* > 0.90 were reliably identified as of Spanish sparrow ancestry, and individuals with *q* < 0.1 were identified as of house sparrow ancestry (Table S4; compare Figures 4 and 5 and Figure S3). However, hybrid classes could not be reliable distinguished from each other and from backcrosses with either of the parental species. Therefore, we generally classified sparrows with assignment probabilities of 0.1 < *q* < 0.90 as of hybrid origin.

In most house sparrow populations, the *P. domesticus* mtDNA lineage and the Z‐chromosomal alleles 1 and 2 were 100% fixed and all individuals were assigned to either of the two house sparrow clusters (Nepal vs. remaining *P. domesticus*) with *q* < 0.1 in microsatellite analysis (Figure 4, Figures S4 and S5). These characteristics were met in the house sparrow populations from Nepal, East Germany, Central Asia, Sudan, and France (in the latter population, a single individual showed a heterospecific allele combination at the *CHD1Z* locus).

In most Spanish sparrow populations, the *P. hispaniolensis* mtDNA lineage and the *CHD1Z* alleles 3–11 were 100% fixed and all individuals were assigned to the Spanish sparrow cluster with *q* > 0.90 in microsatellite analysis. These characteristics were met in the Spanish sparrow populations from Egypt, Central Asia, southern Spain (*q* < 0.90 for a single individual), and Fuerteventura (a single individual showed admixture for the *CHD1Z* locus; Figure 4, Figure S4).

In the area of sympatry on the Iberian Peninsula, 95% PIs of *q*‐values were broader for both parental species (Figure 5b) and almost all house sparrows ranged at slightly higher mean *q* > 0.1 (Figure S3; in contrast, all but one Spanish sparrow individual had a mean *q* > 0.9). We therefore considered this a scenario of weak admixture in the Iberian house sparrow population (similarly, we detected a weak signal of admixture in the Turkish house sparrow population; Figure S3). Furthermore, in southern Spain 10 out of 23 house sparrows carried *CHD1Z* alleles 3 or 6 of Spanish sparrows (whereas the house sparrow allele 1 was absent in Iberian Spanish sparrows Figures 4 and 5b, Figure S4). On the other hand, there was no sign of mitochondrial introgression and no broad overlap of 95% PIs of *q* for the two sympatric species on the Iberian Peninsula (Figure 5b).

All populations of the Italian sparrow showed the same genetic constitution: strong genetic admixture of microsatellite loci, near 100% fixation of *P. domesticus* mtDNA (Figure 4, Figures S2 and S5; except two heteroplasmic individuals from Ustica and Lipari; Figures S5 and S6), and 100% fixation of *CHD1Z* alleles of *P. hispaniolensis* (alleles 3–11; Figure 4, Figures S4 and S6; except one admixed individual on Corsica). Only the population from Lampedusa showed only weak admixture (microsatellites), because most individuals from that island population were assigned to the Spanish sparrow cluster with *q* > 0.90 (Figure 4, Figures S3 and S6).

Patterns of admixture were considerably different in North Africa: All Algerian phenotypic house sparrows, Spanish sparrows, and their hybrids from the mosaic hybrid zone showed strong admixture of mitochondrial and nuclear markers (Figures 4 and 5c, Figure S6). In urban populations, 7%–22% of house sparrows and phenotypic hybrids carried a *P. hispaniolensis ND2* haplotype, whereas in the rural Spanish sparrow population from Hassi El Euch 76% of local individuals carried a *P. domesticus ND2* haplotype (Figures 4 and 5c). Homospecific allele combinations of the sex chromosomal marker (*CHD1Z*: 1/1 = *domesticus* allele; 4/4, 3/3, and 3/4 = *hispaniolensis* alleles) represented more than 50% of the local individual genotypes (Figure S4). In contrast, heterospecific combinations of allele 1 (house sparrow variant) with allele 3 or 4 (Spanish sparrow variant) were underrepresented in the admixed Algerian populations and near absent in populations of the Italian sparrow (only found in one individual from Corsica). West of the mosaic hybrid zone phenotypic house sparrows from Morocco showed a signal of admixture for nuclear markers, whereas in the east a signal of admixture was found in Spanish sparrows from Libya but not in the population from Egypt (Figures S3 and S6).

### 3.2 Diversity and divergence

Algerian populations from the mosaic hybrid zone showed high genetic diversity indices across all three sets of markers (Tables 1 and 2). For instance, populations from the Algerian mosaic hybrid zone showed high values for allelic richness (*CHD1Z*: 3.00 < AR < 4.00; microsatellites 7.50 < AR < 7.90) compared to other Eurasian populations of *P. domesticus* and *P. hispaniolensis* (*CHD1Z*: 1.00 < AR < 2.90; microsatellite: 5.33 < AR < 8.10) and compared to Italian populations of *P. italiae* (*CHD1Z*: 2.00 < AR < 2.75; microsatellite: 6.45 < AR < 7.64) (Tables 1 and 2). Similarly, both nuclear markers showed high values for expected heterozygosity in populations of the Algerian contact zone (*CHD1Z*: 0.58 < *H*_E_ < 0.69; microsatellites 0.84 < mean *H*_E_ < 0.86) compared to Eurasian *P. domesticus* and *P. hispaniolensis* populations (*CHD1Z*: monomorphic, 0.28 < *H*_E_ < 0.56; microsatellite: 0.72 < mean *H*_E_ < 0.86) and *P. italiae* populations (*CHD1Z*: 0.37 < *H*_E_ < 0.54; microsatellite: 0.76 < mean *H*_E_ < 0.85) (Tables 1 and 2). Furthermore, mitochondrial haplotype and nucleotide diversities reflect the same trend, that is, Algerian contact zone populations show high values compared to most Eurasian populations of *P. domesticus*, *P. hispaniolensis*, and *P. italiae* (Table 1). Finally, it is interesting to note that two strictly allopatric populations at the western and eastern range margins of *P. domesticus* (Nepal) and *P. hispaniolensis* (Fuerteventura, Canary Islands) showed least (or low) genetic diversity indices: house sparrows from Nepal (*ND2*: HD = 0.51, *π* = 0.001; micsats: AR = 5.33) and Spanish sparrows from Fuerteventura ( micsats: AR = 5.65; Table 1).

**Table 1:** Diversity indices for mitochondrial ND2 (haplotype diversity = HD, nucleotide diversity = *π* for the total number of haplotypes = *N*_haplotypes_) and 12 microsatellite loci (allelic richness = mean AR, observed and expected heterozygosity = mean *H*_O_ and mean *H*_E_, fixation index = *F*_IS_ [only for samplings *n* ≥ 10], significance level *p < .05, **p < .001)

Taxon (Phenotypic classification)		*N*	Mitochondrial *ND2*			Microsatellites			
			*N*_haplotypes_	HD	*π*	Mean AR	Mean *H*_O_	Mean *H*_E_	*F*_IS_
*P. domesticus* allopatric	Kathmandu (Nepal)	19	4	0.509	0.0010	5.329	0.708	0.740	0.044
	Saxony (East Germany)	17	7	0.713	0.0019	7.432	0.794	0.834	0.050*
	Landes (France)	12	5	0.692	0.0013	7.263	0.797	0.839	0.052
	Kefalonia Island (Greece)	4	2	0.500	0.0007	–	0.646	0.784	–
*P. domesticus* sympatric	Muğla (Turkey)	31	14	0.869	0.0023	8.055	0.747	0.854	0.127**
	Sevilla (Spain)	24	9	0.795	0.0026	7.860	0.795	0.858	0.075**
	Sudan	4	1	0	0	–	0.521	0.780	–
	Central Asia	4	4	0.800	0.0015	–	0.660	0.792	–
	Morocco	4	5	0.933	0.0028	–	0.715	0.806	–
*P. domesticus* Hybrids Algerian contact zone	Djelfa urban	44	14	0.777	0.009	7.956	0.797	0.853	0.066**
	Hassi El Euch urban	19	11	0.865	0.007	7.999	0.773	0.857	0.100**
*P. hispaniolensis* allopatric	Fuerteventura Island (Spain)	19	10	0.869	0.0027	5.648	0.668	0.723	0.078*
	Giza (Egypt)	6	3	0.524	0.0008	–	0.833	0.826	–
	Libya	4	3	1.000	0.0030	–	0.775	0.803	–
*P. hispaniolensis* sympatric	Sevilla (Spain)	23	10	0.710	0.0017	7.346	0.786	0.811	0.031
	Central Asia	3	1	0	0	–	0.861	0.822	–
*P. hispaniolensis* Algerian contact zone	Hassi El Euch rural	25	9	0.625	0.0164	7.498	0.802	0.837	0.043
*P. italiae*	Sicily East, Maletto (Italy)	11	2	0.182	0.0003	7.644	0.825	0.814	−0.015
	Sicily West, Fraginesi (Italy)	10	2	0.200	0.0003	7.142	0.787	0.795	0.011
	Lampedusa Island (Italy)	14	3	0.362	0.0006	6.446	0.756	0.786	0.040
	Ustica Island (Italy)	10	2	0.222	0.0003	6.964	0.829	0.828	0.000
	Lipari Island (Italy)	5	2	0.500	0.0223	–	0.817	0.852	–
	La Chiappa, Corsica (France)	4	2	0.667	0.0010	–	0.792	0.759	–
	Pantelleria Island (Italy)	4	3	0.833	0.0015	–	0.729	0.802	–

**Table 2:** Diversity indices for the Z‐chromosomal marker *CHD1Z* calculated for the total number of alleles

Taxon (Phenotypic classification)		*N* spec	*N* seq	AR	*H*_O_	*H*_E_	HWE *p* value	FIS	*π* (JC)
*P. domesticus*	Norway^a^	14	28	1.0	Monomorph	Monomorph	–	Fixed	0
	Saxony (East Germany)	7	14	1.0	Monomorph	Monomorph	–	Fixed	0
	Sevilla (Spain)	21	42	2.89	0.191	0.556	*p* < .00001	0.663 *p* = .005	0.00289
*P. domesticus* Hybrids Algerian contact zone	Djelfa urban	38	76	3.00	0.421	0.675	.0098	0.379 *p* = .005	0.00355
	Hassi El Euch urban	7	14	4.00	0.429	0.582	.2248	0.280 *p* = .22	0.00212
*P. hispaniolensis*	Pula, Sardinia (Italy)^a^	16	32	2.35	0.313	0.280	*p* = 1.0	−0.119 *p* = 1.0	0.0075
	Sevilla (Spain)	11	22	2.00	0.364	0.416	*p* = 1.0	0.130 *p* = .575	0.00108
*P. hispaniolensis* Algerian contact zone	Hassi El Euch	25	50	3.55	0.480	0.693	.0450	0.312 *p* = .030	0.00349
*P. italiae*	Southern Italy [Our data]	15	30	1.99	0.200	0.370	.1300	0.468 *p* = .1150	0.00097
	Central Italy [Acquaviva‐Picena^a^]	14	28	2.75	0.607	0.542	.7045	−0.124 *p* = .810	0.00157

Note

Males only: allelic richness (AR), observed and expected heterozygosity (*H*_O_, *H*_E_), fixation index (*F*_IS_); *N*spec = number of specimens, *N*seq = number of sequences.

a Populations from Elgvin et al. (2011), sequence data from GenBank.

*F*_ST_ values inferred from microsatellite data (12 loci) were not significantly different from 0 for most pairwise comparisons of admixed urban populations at Djelfa and Hassi El Euch with most other study populations of all three species (Table 3). Moreover, pairwise F_ST_ values were generally highest for comparisons of the Himalayan *P. domesticus* population from Nepal with all other study populations (0.109 < *F*_ST_ < 0.212). Remarkably, pairwise F_ST_ values for intraspecific comparisons of Himalayan and Western Palearctic house sparrow populations were higher (0.109 < *F*_ST_ < 0.124) than those for interspecific comparisons between Western Palearctic house sparrows and Spanish sparrows (e.g., from Spain; 0.024 < *F*_ST_ < 0.099; Table 3).

**Table 3:** Pairwise *F*_ST_ Values, for 16 study populations (*n* > 5) based on 12 microsatellite loci, lower diagonal; Bonferroni corrected (alpha = 0.06/120 = 0.0004); significant *F*_ST_ Values are in bold

Species	Locality	*P. domesticus* Allopatric			*P. domesticus* Sympatric		*P. domesticus*, Hybrids Algerian contact zone				*P. hispaniolensis*			*P. italiae*			
											Allop.	Symp.	Contact				
		1	2	3	4	5	6	7	8	9	10	11	12	13	14	15	16
*P. domesticus* Allopatric	(1) Kathmandu (Nepal)																
	(2) Dresden (Germany)	**0.124**															
	(3) Landes (France)	**0.119**	−0.006														
*P. domesticus* Sympatric	(4) Muğla (Turkey)	**0.109**	0.003	0.015													
	(5) Sevilla (Spain)	**0.117**	−0.003	−0.001	0.011												
*P. domesticus*, Hybrids Algerian contact zone	(6) Djelfa (*domesticus*)	**0.129**	0.011	0.010	0.014	0.003											
	(7) Djelfa (hybrids)	**0.128**	0.012	0.005	0.019	0.005	0.007										
	(8) Hassi El Euch (*domesticus*)	**0.136**	0.007	0.017	0.014	0.010	0.001	0.005									
	(9) Hassi El Euch (hybrids)	**0.146**	0.028	0.027	0.025	0.005	0.000	0.012	0.014								
*P. hispaniolensis* Allopatric	(10) Fuerteventura Island (Spain)	**0.212**	**0.094**	**0.098**	**0.084**	**0.063**	**0.043**	**0.061**	**0.071**	0.029							
*P. hispaniolensis* Sympatric	(11) Sevilla	**0.166**	**0.044**	**0.045**	**0.044**	**0.024**	0.012	**0.024**	**0.028**	0.012	**0.030**						
*P. hispaniolensis,* Algerian contact zone	(12) Hassi El Euch (*hispaniolensis*)	**0.130**	**0.025**	**0.025**	**0.020**	**0.015**	0.003	0.011	0.009	0.000	**0.044**	**0.017**					
*P. italiae*	(13) Sicily East (Maletto)	**0.178**	**0.053**	**0.048**	**0.045**	**0.035**	0.017	0.022	0.044	0.016	**0.054**	0.010	0.010				
	(14) Sicily West (Fraginesi)	**0.175**	**0.036**	**0.038**	**0.033**	0.022	0.010	0.017	0.020	0.010	**0.049**	0.013	0.010	0.012			
	(15) Lampedusa Island (Italy)	**0.181**	**0.054**	**0.062**	**0.061**	**0.044**	**0.045**	**0.044**	**0.044**	0.042	**0.060**	**0.032**	**0.039**	**0.044**	0.042		
	(16) Ustica Island (Italy)	**0.148**	0.018	0.011	**0.029**	0.009	0.015	0.014	0.028	0.027	**0.076**	0.027	0.023	0.035	0.026	**0.040**	

### 3.3 Phenotypic variation

**Figure 6:**
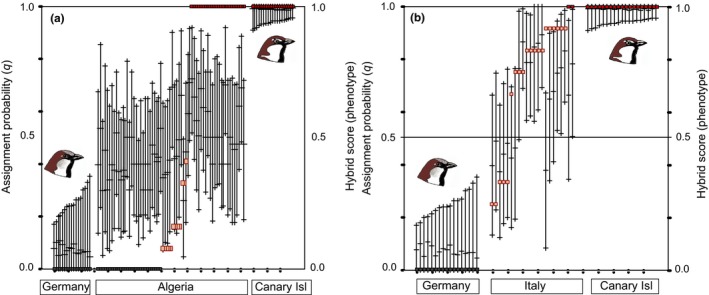
Phenotypic and genetic admixture in North African hybrid populations (a) and the Italian hybrid form *Passer italiae* (compared to allopatric populations of the parental species *Passer domesticus* [Germany] and *Passer hispaniolensis* [Fuerteventura, Canary Islands]); assignment probabilities: bars = individual 95% probability intervals of *q*; individual phenotypes, hybrid scores from 0 = *P. domesticus* (gray boxes) to 1 = *P. hispaniolensis* (brown boxes); intermediate phenotypes 0 < score < 1 (light beige boxes)

In Algeria, urban populations showed highly variable combinations of the six plumage color traits (Figure 3, Table S1). All urban birds had at least traces of gray in the crown. However, phenotypic house sparrows were overrepresented in urban study populations (72.5% of the total sampling; hybrid score of 0, that is, six of six characters matching the house sparrow phenotype). In the rural population from Hassi El Euch, only *P. hispaniolensis* phenotypes were found (100% had the hybrid score 1, that is, six of six characters matched the Spanish sparrow phenotype). None of the birds from that crop field population had gray in the crown: Most birds showed the characteristic brown crown of *P. hispaniolensis*, although some birds had an aberrant phenotype with a black central crown patch (Figure 3G). Hybrid scores and genetic admixture proportions did not coincide in the Algerian study populations, because 95% PIs of *q* showed large overlap even between phenotypic *P. domesticus* and phenotypic *P. hispaniolensis* (Figure 6a). In contrast, island populations of *P. italiae* showed a greater match between genetic admixture proportions and phenotypic hybrid scores (Figure 6b); for example, populations from Sicily and Lampedusa were phenotypically and genetically more similar to *P. hispaniolensis*, whereas the population from Ustica was more similar to *P. domesticus* (Figure 6, Figures S3 and S5).

## 4 DISCUSSION

In this study, we have confirmed several patterns of divergence and admixture previously described for Eurasian continental sparrow populations. Allopatric populations of house sparrows and Spanish sparrows could be clearly identified and assigned to separate lineages of the mtDNA and the sex chromosomal marker and to separate clusters in the microsatellite analysis (in accordance with Elgvin et al., 2011; Hermansen et al., 2011). Interestingly, our results even confirmed intraspecific differentiation of the house sparrow with respect to genetic distinctiveness of Asian populations (Ravinet et al., 2018). In the Spanish sparrow, the population from the Canary Islands showed greatest divergence values (Ravinet et al., 2018). Thus, the two most peripheral populations of our sampling were the most genetically diverged ones. Genetic divergence of populations at the periphery of a wide continental range typically arises from drift and founder effects that act most strongly on small and isolated populations (reviewed in: Hardie & Hutchings, 2010; birds: Kvist, Arbabi, Päckert, Orell, & Martens, 2007; Päckert, Martens, Hering, Kvist, & Illera, 2013; other terrestrial vertebrates: Fritz, Barata, Busack, Fritzsch, & Castilho, 2006; Schwartz, Mills, Ortega, Ruggiero, & Allendorf, 2003).

Our results furthermore indicate that interspecific introgression of nuclear markers increases with the proximity of a population to the area of sympatry (Ravinet et al., 2018; for Iberian populations compare Hermansen et al., 2014). However, these findings do not disprove reproductive isolation between the two parental species, but the near absence of phenotypic hybrids in the Eurasian area of sympatry rather suggests that despite limited interspecific gene flow, species integrity of these two is maintained due to genomic parental incompatibilities (Elgvin et al., 2017; Hermansen et al., 2014; Trier et al., 2014). In contrast, in the North African mosaic hybrid zone strong genetic admixture despite spatial, temporal, and ecological separation of phenotypic house sparrows, Spanish sparrows, and their hybrids is an unexpected striking result.

On the one hand, cautious interpretation of admixture proportions inferred from microsatellites has been recommended for a number of reasons. Pitfalls associated with the use of short tandem repeats (STRs) for population genetic studies relate to their particular mechanism of mutation (Putman & Carbone, 2014), a generally assumed inferiority of neutral markers compared to functional markers (Liebl, Schrey, Andrew, Sheldon, & Griffith, 2015), possible overestimate of gene flow (Balloux, Brünner, Lugon‐Moulin, Hausser, & Goudet, 2000; Balloux, Lugon‐Moulin, & Hausser, 2000), and to the fact that STRs do not represent genome‐wide variation as, for example, inferred from single nucleotide polymorphisms (SNPs) (Lemopoulus et al., 2019). On the other hand, several comparative studies have concluded that despite all limitations, microsatellite data are not generally less informative or less suitable for detection of patterns of divergence and admixture than genome‐wide data, for example, SNPs (Fernández et al., 2013; Ljungqvist, Åkeson, & Hansson, 2010; Narum et al., 2008; Roques, Chancerel, Boury, Pierre, & Acolas, 2019). Moreover, we stress that in our sparrow data set, there is a considerable difference between patterns of admixture in Algeria compared to the situation in the European area of sympatry where only limited asymmetrical allelic introgression occurs (i.e., on the Iberian Peninsula: this study in accordance with Hermansen et al., 2014). That particular situation in North Africa thus requires an explanation.

### 4.1 Strong genetic admixture despite prezygotic barriers in North Africa

First, the North African mosaic hybrid zone is considered a very recent phenomenon (Cramp & Perrins, 1994: p. 320). Early historical field explorations suggested a recent eastward colonization of the Maghreb by the house sparrow during the 2nd half of the 19th century when house sparrows were still absent in many parts of Algeria (extensive review in Glutz von Blotzheim & Bauer, 1997: p. 42; Heim de Balsac & Mayaud, 1962: p. 390/391; Rothschild & Hartert, 1912). Further eastward expansion of the mosaic hybrid zone has been documented when hybrid populations dispersed to previously unsettled parts of Algeria east of 2°E and Tunisia from the first decades of the 20th century (Summers‐Smith & Vernon, 1972). Thus, regular colonization of new habitats and new formation of locally admixed sparrow assemblages might have promoted admixture of local gene pools until recently but prevented the emergence of a stabilized hybrid form like on the Italian Peninsula (Elgvin et al., 2011; Hermansen et al., 2011). Yet, the relatively recent establishment of sympatry is probably not the sole explanation for a high degree of genetic admixture in North African sparrow populations, because on Gargano Peninsula (Italy) where Spanish sparrows were introduced less than a decade ago local sympatry with the Italian sparrow did not result in any pattern of admixture despite differences in habitat preferences and breeding phenology (Sætre et al., 2017).

Second, genetic admixture was not only found in phenotypic house sparrows from the Algerian contact area but also for the Moroccan house sparrow population (*Passer domesticus tingitanus*). In northwest Africa, genetically admixed house sparrow populations might have originated from (a) local hybridization with Moroccan Spanish sparrows or (b) ancestral polymorphism, because colonization of North Africa by the house sparrow was suggested to have occurred via the Strait of Gibraltar from Iberian source populations (Summers‐Smith, 1988; supported by *ND2* haplotype dom3 that was found only in Iberian and Moroccan house sparrow populations, Figure S5). In both scenarios, the North African mosaic hybrid zone would have been formed by range expansion of genetically admixed populations of the invasive parental species, the house sparrow (*P. d. tingitanus*).

Third, the unique spatial distribution pattern of North African sparrow populations might offer an alternative explanation. According to the desperation hypothesis (Hubbs, 1955), the probability for heterospecific matings and hybridization should be strongly increased in numerically imbalanced populations (McCracken & Wilson, 2011). Following this reasoning, it has been assumed that excessive interbreeding among house sparrows and Spanish sparrows was limited to those areas like North Africa where either of the two parents is rare (Summers‐Smith, 1988; Hermansen et al., 2011; similarly for hybrids of *P. domesticus* × *Passer montanus* in Belgium: Bronne, 2009). Indeed, due to spatial separation the two parental sparrow species would only come into contact in the outskirts of villages, where house sparrows are indeed largely underrepresented in numbers (Figure 2a) and will face occasional encounters with Spanish sparrows while foraging in the crop fields. In this scenario, extant hybridization might then occur via regular heterospecific matings, including a certain level of extra‐pair paternity (EPP). EPP is common in several bird species (Griffith, Owens, & Thuman, 2002; Randler, 2008) and has been reported in both house sparrows and Spanish sparrows (Bichet et al., 2014; Møller, 1987; Summers‐Smith, 1954). Moreover, EPP has been considered a driver of sexual selection and asymmetrical introgression in hybrid zones (Baldassare & Webster, 2013; Svedin, Wiley, Veen, Gustafsson, & Qvarnström, 2008) and its extent depends on a number of factors such as mating system and social structure of the species involved (Hartmann, Wetzel, Crowley, & Westneat, 2011; Hasselquist & Sherman, 2001). Because of the similar female phenotypes of *P. domesticus* and *P. hispaniolensis*, the study of sparrow mating systems and possible heterospecific matings in areas of sympatry in the wild will be a challenging task for future field research.

### 4.2 Parental phenotype integrity despite genetic admixture in North Africa

Though admixed urban populations in the Algerian study area showed a high phenotypic variation, we found a strong spatial separation of parental phenotypes. Despite generally strong genetic admixture, the majority of local breeders in the cities showed the house sparrow phenotype and 100% of the rural population showed the Spanish sparrow phenotype. The main reason for this phenotype–genotype discordance is certainly the impact of selection not only on plumage color traits but also on biometric traits in sparrows. Evolutionary constraints on biometric traits may differ among sexes (e.g., in the house sparrow: Jensen et al., 2003). In particular, beak size and shape were suggested to be under divergent selection (Eroukhmanoff et al., 2013; Runemark, Fernández, et al., 2018) whereas bill length is likely to be subject to epigenetic modifications (Riyahi et al., 2017). Accordingly, in Algerian study populations phenotypic house sparrows and Spanish sparrows could be clearly distinguished by biometric analysis, whereas hybrids were more similar to house sparrows (Belkacem et al., 2016). Among plumage color traits, crown color seems to be under strongest divergent selection (Runemark, Fernández, et al., 2018). For example, in the alpine hybrid zone between the house sparrow and the Italian sparrow crown color showed a strongly bimodal distribution and the narrowest cline of three plumage traits (along with color of cheek and supercilium; Bailey et al., 2015). Furthermore in different populations of the Italian sparrow, different plumage color traits have evolved back toward different parental phenotypes resulting in distinctive local trait mosaicism that was strongest on the islands of Crete and Corsica (Runemark, Fernández, et al., 2018; compare different mosaic phenotypes on Ustica, Sicily, and Lampedusa in our data set; Figure S2). Accordingly, Runemark, Fernández, et al. (2018) suggested a high novelty potential for traits under divergent selection, such as crown color in sparrows. Indeed, we could document novel trait variation in Algerian Spanish sparrows having an intensely black crown (Figure 3G). That color variation does not occur elsewhere across the breeding range of *P. hispaniolensis* (Cramp & Perrins, 1994), but has been documented already by Rothschild and Hartert (1912) in Algeria. So that particular variant of black‐crowned Spanish sparrows has now persisted in the North African mosaic hybrid zone for at least roughly a hundred years.

## 5 CONCLUSIONS

The outcome of hybridization may not solely result from the degree of genetic (in)compatibility between genomes of parental species, that is, postzygotic reproductive barriers that strengthen over divergence time. Instead, additional factors can be important such as prezygotic reproductive barriers (e.g., behavior, life cycle, gamete compatibility) or environment‐dependent selection regimes acting on parental species and their hybrids. If the complex interplay between these factors varies over time and space, spatial patterns in secondary contact can be very different and range from sharply defined narrow hybrid zones to patchy distributions of parental species and hybrid populations in mosaic hybrid zones (Curry, 2015; Jiggins & Mallet, 2000). In fact, there are well‐studied examples where secondary contact between the same species resulted in different patterns of admixture at different localities, such as in marine invertebrates (*Mytilus edulis*, *Mytilus trossulus*: Riginos & Cunningham, 2005; Stuckas et al., 2017) or tortoises (*Mauremys*: Vamberger et al., 2017). The sparrow hybrid system provides another striking case where differentially structured hybrid zones between the same species exist in different regions of their range of overlap.

Postglacial range expansion of the house sparrow was presumably strongly associated with the rise of human agriculture and civilization and adaptation to novel habitat in an anthropogenic environment (Sætre et al., 2012). Likewise, the very recent formation of the North African hybrid zone went along with historical dispersal of house sparrows into Algeria that coincides with increasing urbanization of that region in the late 19th century (Hadjri & Osmani, 2004; Summers‐Smith & Vernon, 1972). Hybridization with the Spanish sparrow might then have promoted further eastward range expansion of the house sparrow into novel (anthropogenic) habitats as postulated for other species (Pfennig, Kelly, & Pierce, 2016; Pierce, Guitierrez, Rice, & Pfennig, 2017; Seehausen, 2004). In North Africa, this seems to be an ongoing process promoted by intensification of agriculture and cultivation of new crop fields during recent decades including massive recent dispersal of hybrid sparrows even to hyper‐arid regions where large hybrid colonies exist in the absence of either of the parental species (Guezhoul, Chenchouni, & Doumandji, 2011; Guezhoul et al., 2013). Similar effects of very recent human‐mediated range expansions and intensified urbanization have been reported from other parts of the house sparrow's range (Schrey, Liebl, Richards, & Martin, 2014; Sheldon et al., 2018; Vangestel et al., 2012). Generally, anthropogenic disturbances might facilitate hybridization between ecologically divergent species that under different (undisturbed) conditions do not regularly interbreed (“anthropogenic hybridization”: See Grabenstein & Taylor, 2018; McFerlane & Pemberton, 2019). In all these aspects, the admixed North African sparrow populations are in accordance with the “mosaic hybrid zone model” characterized by a spatial patchwork of secondary contact and possible “escape” of hybrids to new habitats under certain local environmental conditions (Curry, 2015).

## CONFLICT OF INTEREST

None declared.

## AUTHOR CONTRIBUTIONS

MP, AAB, and HS designed the theoretical and methodical framework. HW and OG performed laboratory work and most statistical analyses. MP and MV performed further statistics. GG and MLV carried out fieldwork and collected samples in Italy, AAB in Algeria, DC in Spain, and JM in Nepal and other regions of Asia. MW provided further samples. MP drafted the manuscript. All authors read the draft and contributed to the discussion and completion of the final manuscript.

## Data Availability

*ND2* sequences from Belkacem et al. (2016) used for this study are available at GenBank under accession nos. KX370619–KX370815. *ND2* and *CHD1Z* sequences that were newly generated for this study have been submitted to GenBank (accession nos. *ND2*: MN488840–MN488995, *CHD1Z*: MN489495–MN490043). A data package including microsatellite allele lengths, a full material with locality information, and alignments of *ND2* and *CHD1Z* was deposited in the Dryad Digital Repository with https://doi.org/10.5061/dryad.v9s4mw6qf.
