# Supplementary material for: Genetic admixture despite ecological segregation in a North African sparrow hybrid zone (Aves, Passeriformes, Passer domesticus × Passer hispaniolensis)
Source: Ecol Evol. 2019 Oct 28;9(22):12710–26. doi: 10.1002/ece3.5744 (PMC6875665; doi:10.1002/ece3.5744)
Supplement: Supplementary file 1 [file ECE3-9-12710-s001.docx]

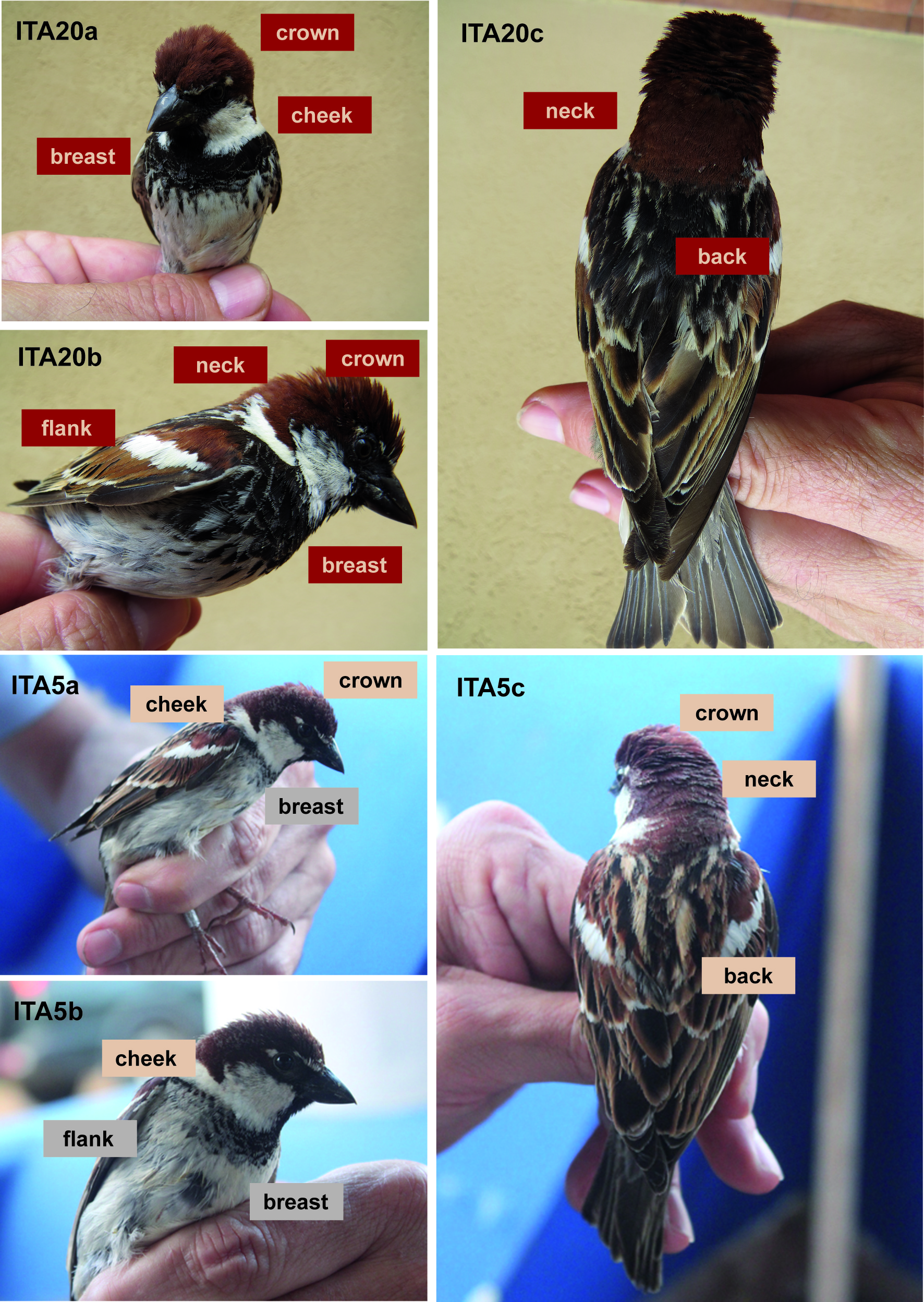


Fig. S1: Phenotypic variation among island populations of *Passer italiae*; individual ITA20 from Fraginesi (western Sicily) showed the parental *P. hispaniolensis* phenotype in all six plumage traits and thus scored a hybrid index of 1. In contrast, individual ITA5 (that was heteroplasmic for the mtDNA marker ND2) had the breast and flank color pattern of the house sparrow (*P. domesticus*) and all other plumage traits intermediate (e.g. grey at crown and neck feather tips). Accordingly, it received a hybrid score of 0.167.


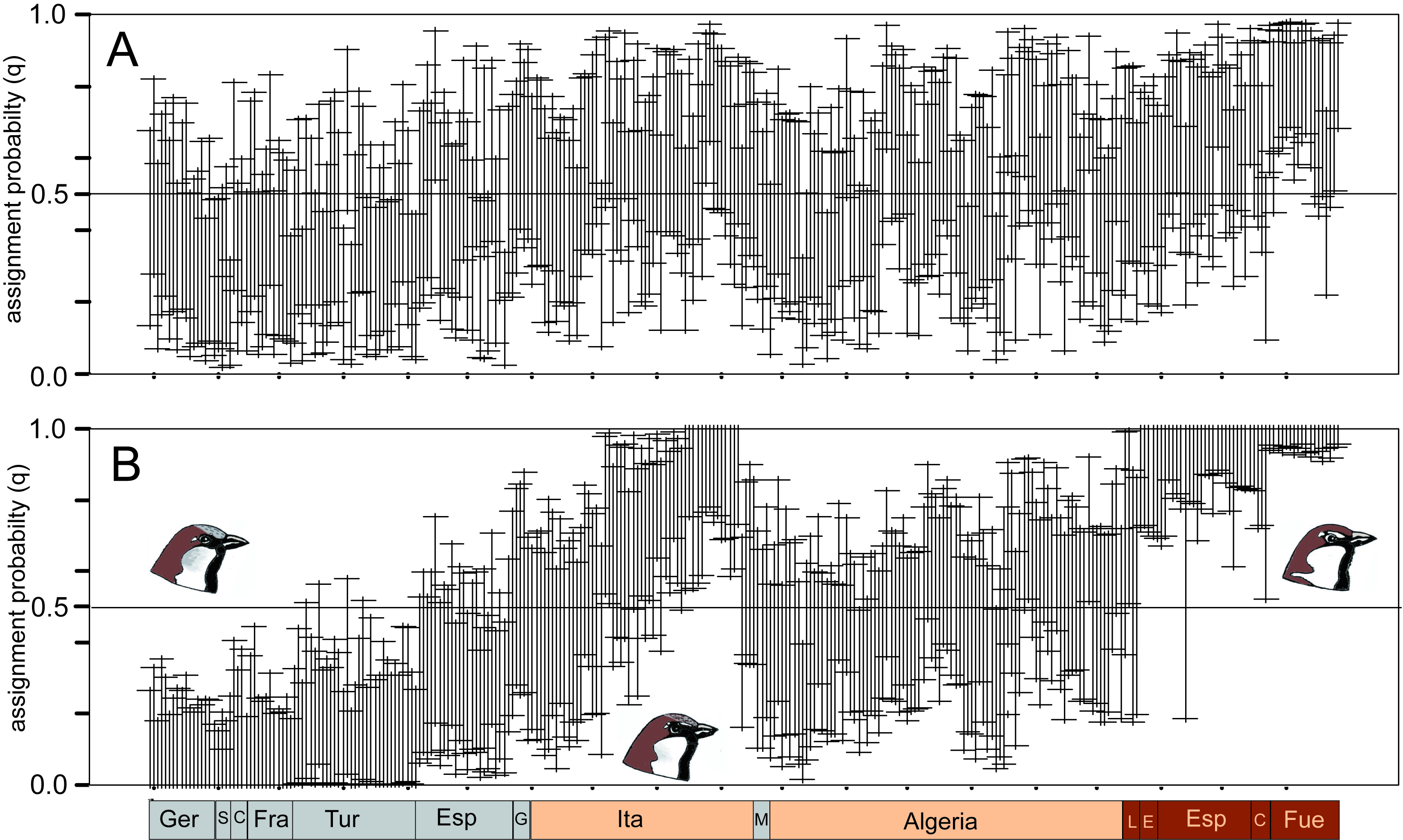


**Figure S2**: 95% probability intervals for q from STRUCTURE runs with admixture model and correlated allele frequencies for the reduced data set excluding the population from Nepal (301 samples, K=2); A) without locpriors, B) with population locprior; species and populations indicated on bars below plots (Algerian populations labelled as admixed [light beige] regardless of local phenotypes): house sparrow populations (*P. domesticus*)= grey: East German (Ger), Sudan (S), Central Asia (C), France (Fra), Turkey (Tur), Spain (Esp), Greece (G), Morocco (M); Italian sparrows (*P. italiae*)= light beige; Spanish sparrow populations= brown: Libya (L), Egypt (E), Spain (Esp), Central Asia (C), Fuerteventura (Fue).


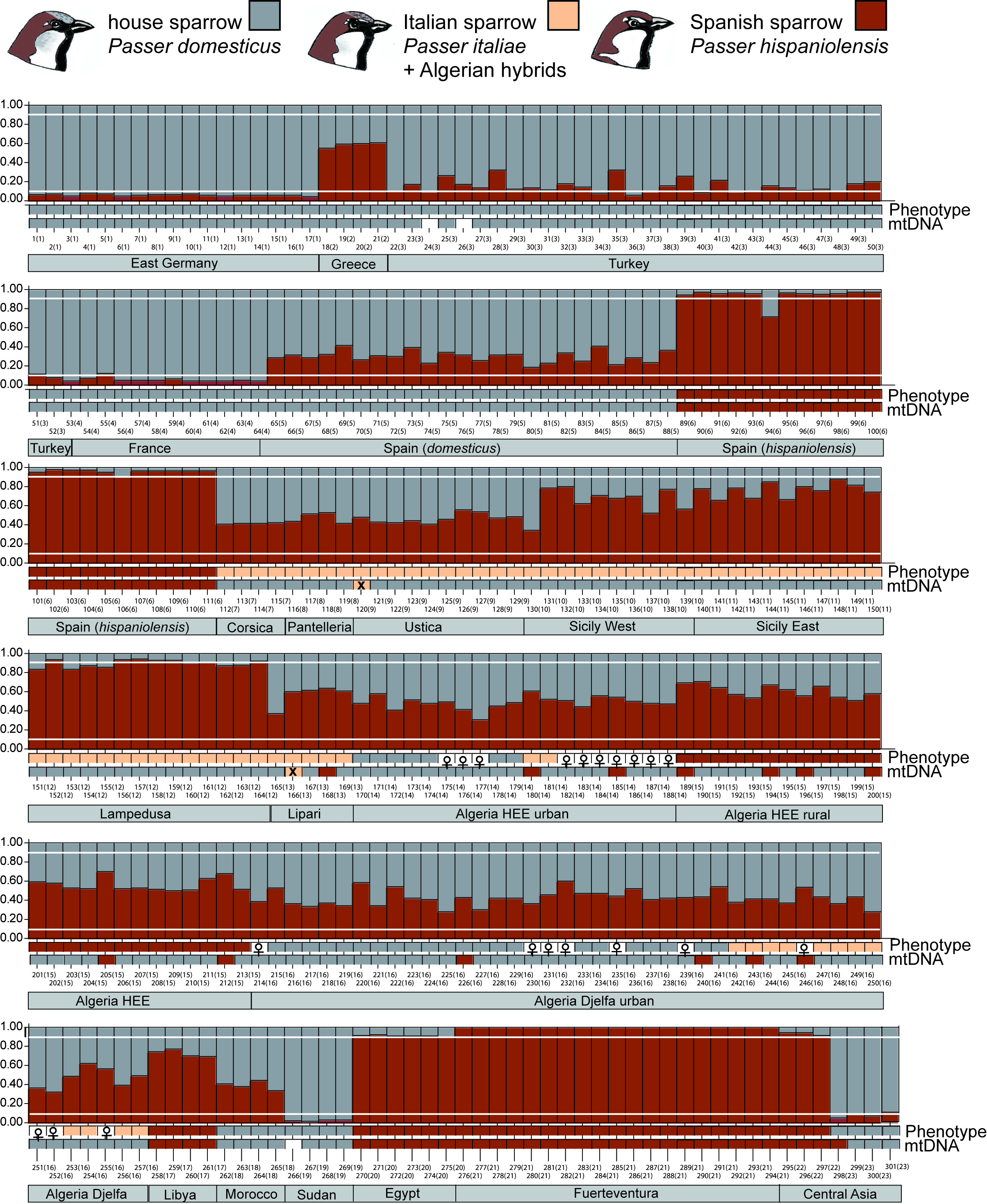


**Figure S3**: STRUCTURE plot for k=2, reduced data set under exclusion of the Himalayan *P. domesticus* population from Nepal; each bar represents one individual with bar coloration indicating genetic admixture proportions to either *P. domesticus* (grey) or *P. hispaniolensis* (brown); individual phenotypes (light beige) and mtDNA haplotype lineages assigned below the respective bars; females from Algerian study sites were not assigned to a particular phenotype of the sympatric parental and hybrid populations (left blank and marked with symbol), whereas for example all individuals from the Italian study populations (males, females, juveniles) were classified as phenotype “*P. italiae*”; two heteroplasmic Italian sparrows (*P. italiae*; ITA5 and ITA52) carrying mtDNA of both parental species indicated by “x”.


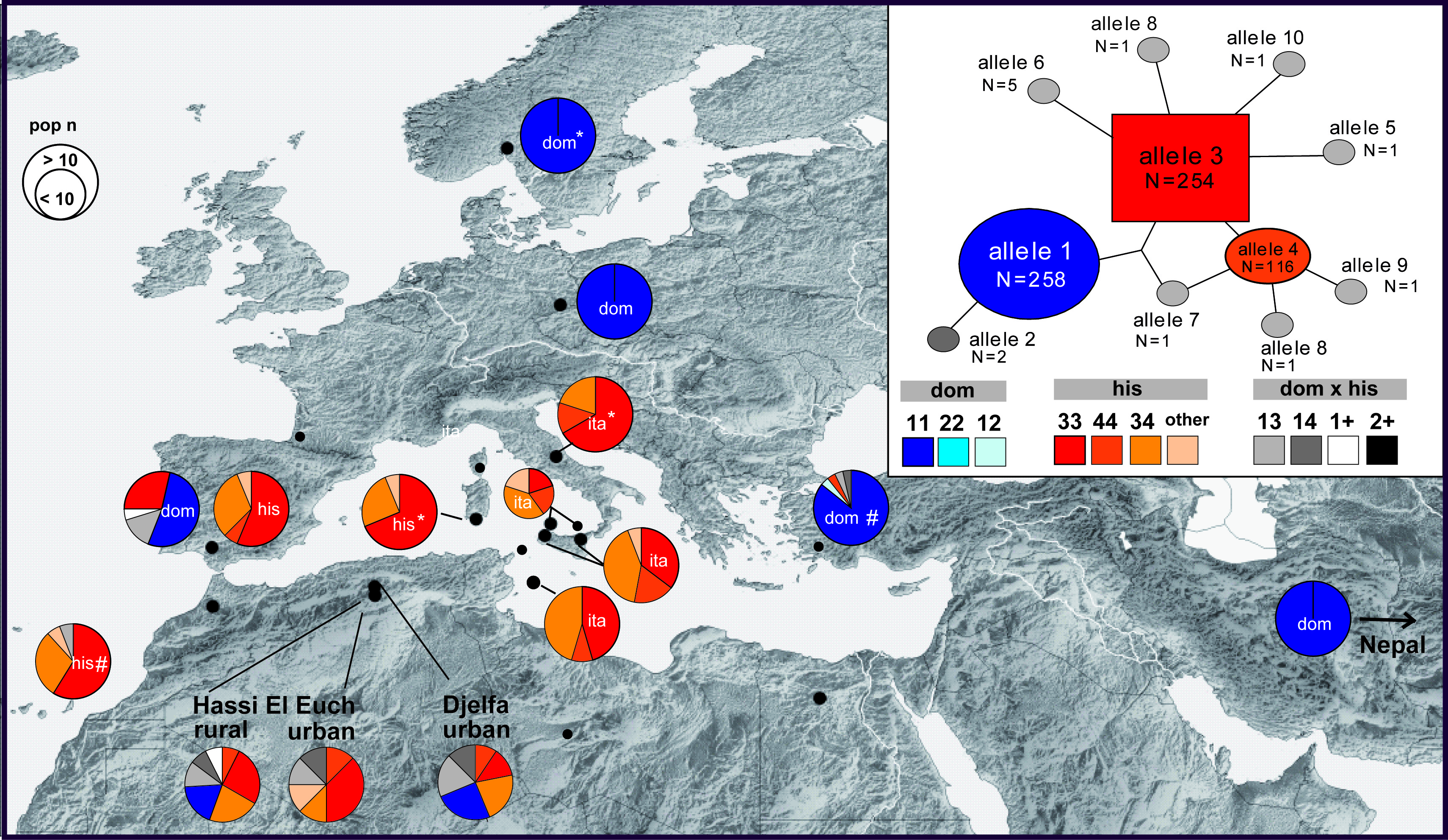


**Figure S4**: Geographic variation of the z-chromosomal *CHD1Z* locus; haplotype network for eleven distinct alleles, upper right; local allele frequencies in study populations indicated by pie charts, males only (except two populations with limited information on sex identification marked by “#”: *P. hispaniolensis* from Fuerteventura, *P. domesticus* from Turkey); phenotypic assignment of study populations indicated by dom= *P. domesticus*, his= *P. hispaniolensis*, ita= *P. italiae*, hyb= North African hybrids; *= study populations from Elgvin et al. (2011), sequence data inferred from GenBank.


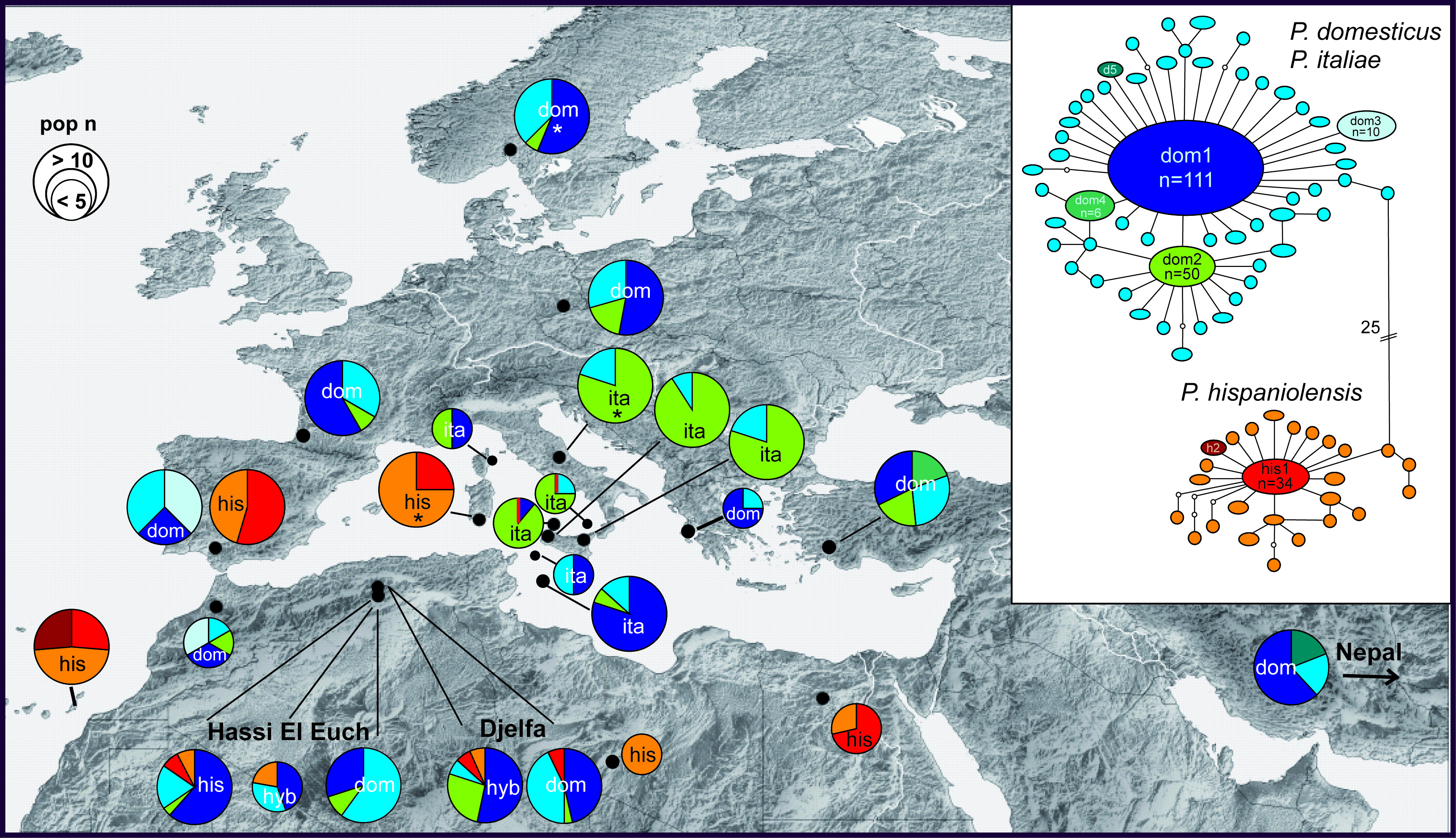


**Figure S5**: Differentiation and geographic variation of mitochondrial DNA (ND2) in house sparrows, Spanish sparrows, Italian sparrows and Algerian hybrid populations (including sequence data sets from Norway, Central Italy and Sardinia by Hermansen et al. 2011; marked by an asterisk); upper right: minimum spanning network (674 bp); presence of *P. hispaniolensis* mtDNA in two insular populations of *P. italiae* due to paternal leakage indicated by red vertical bar in the pie charts.


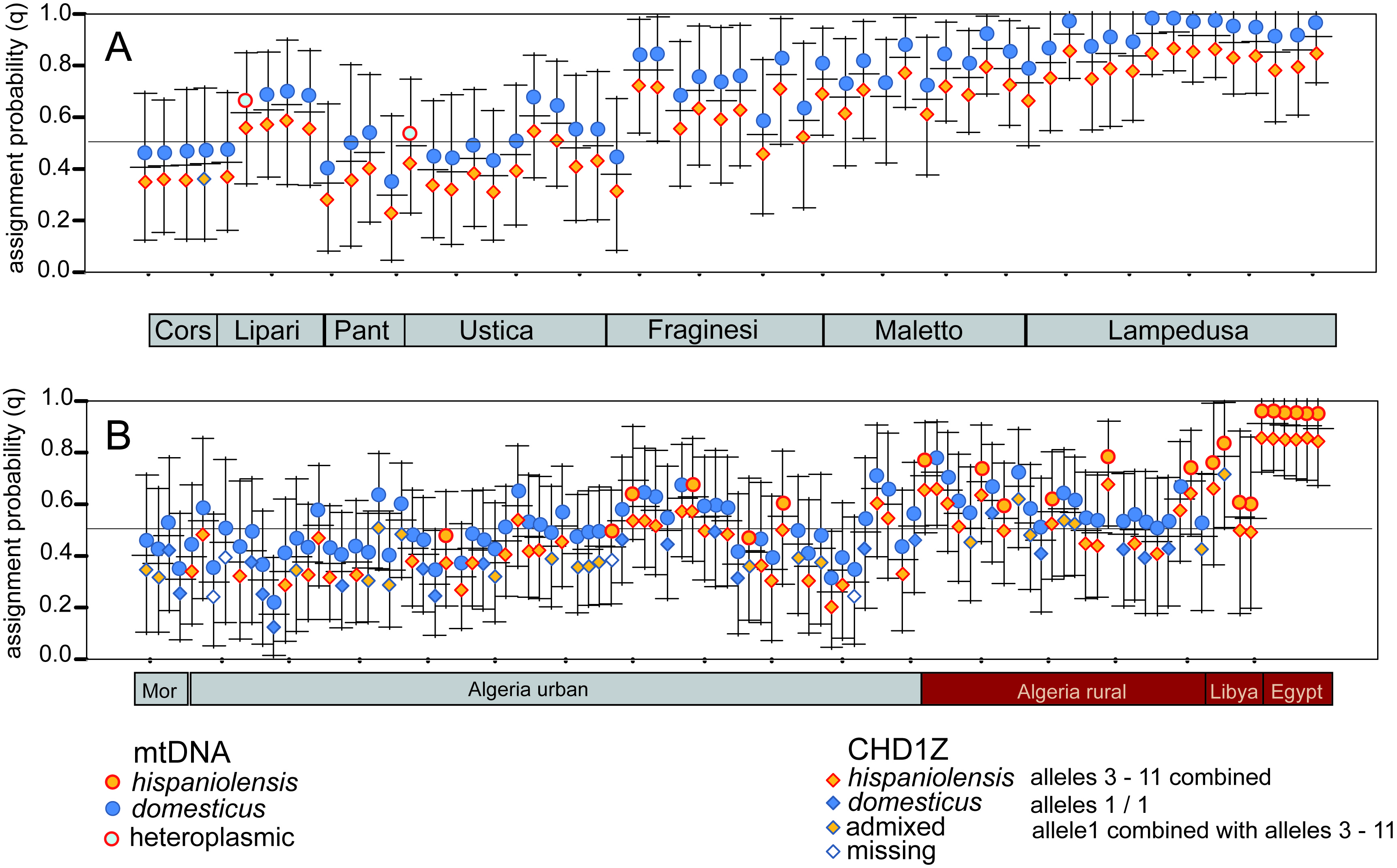


**Figure S6**: Patterns of admixture in A) island populations of the Italian sparrow (*P. italiae*) and B) in the North African mosaic hybrid zone compared to house sparrows (*P. domesticus*) in the West (Morocco= Mor) and Spanish sparrows (*P. hispaniolensis*) in the East (Lybia and Egypt); each line represents an individual’s 95% PI for q-values (13 microsatellite loci; assignment to the Spanish sparrow cluster relative to the house sparrow cluster; in ascending order from 0= house sparrow ancestry to 1= Spanish sparrow ancestry); ND2 haplotypes of each individual indicated by colored dots; CHD1Z allele combinations for each individual indicated by colored diamonds.
