# Supplementary material for: Genetic admixture despite ecological segregation in a North African sparrow hybrid zone (Aves, Passeriformes, Passer domesticus × Passer hispaniolensis)
Source: Ecol Evol. 2019 Oct 28;9(22):12710–26. doi: 10.1002/ece3.5744 (PMC6875665; doi:10.1002/ece3.5744)
Supplement: Supplementary file 2 [file ECE3-9-12710-s002.docx]

**Table S1: Classification of parental species and hybrid phenotypes, hybrid score (from 0 to 1) according to six plumage color traits**; pie charts at the right show composition of Algerian study populations from the study period (years 2010/2011) compared to data from an earlier field season (year 2006) from the same region but different study sites; from the year 2006 field season 17 individuals with one parental plumage color trait (and five intermediate traits) were listed. However, there was no distinction between hybrid classes “his5” and “dom5”, so we just treated these 17 individuals as pooled hybrid class 5.

**
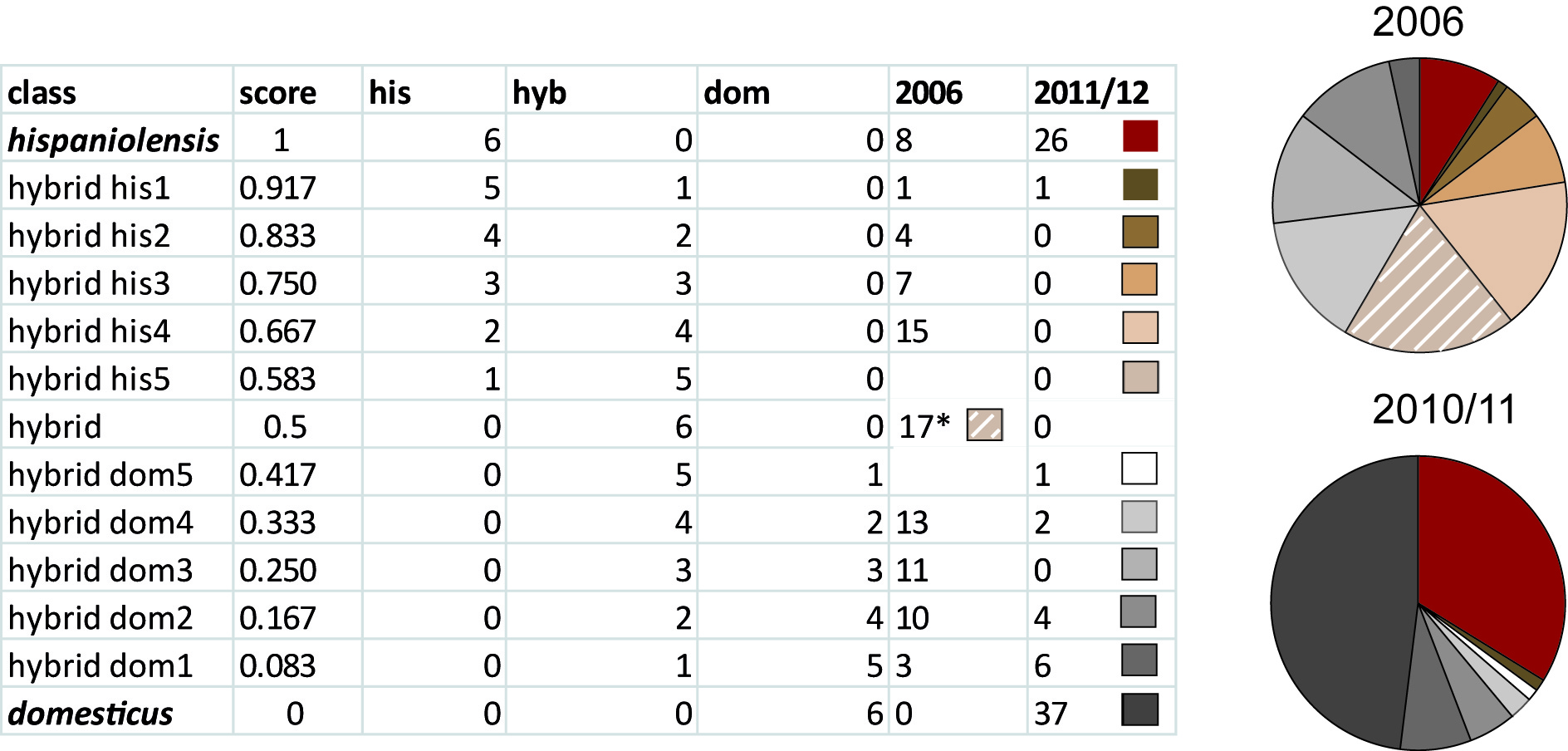
**

**Table S2: Basic information about the microsatellite loci used in this study.** F: is the forward primer, R: the reverse primer, Tm is the locus specific melting temperature and the 5´-modification is the respective fluorescence label of the forward primer.

| **Locus** | **Source species** | **Reference** | **Accession no.** | **Repeat motif** | **Primer sequence 5´-3** | **T_m_ (°C)** | **5´-modification (fluro label)** | **multiplex** | **allele size, exp. (bp)** | **allele size, obs. (bp)** |
| --- | --- | --- | --- | --- | --- | --- | --- | --- | --- | --- |
| Pdo 1 | *P. domesticus* | Neumann (1996) | AM287188 | (GT)_23_ | F:TCTGGGCTGTTGTCAGAAGGA | 59 | 6-FAM | B | 158 - 199 | 150 - 209 |
|  |  |  |  |  | R:GCAGGGCTGTCCTTTCAAACT | 60 |  |  |  |  |
| Pdo 3 | *P. domesticus* | Neumann (1996) | AM287190 | (ATCC)_17_ | F:CTGTTCATTAACTCACAGGT | 41 | Atto 550 | A | 118 - 172 | 108 - 166 |
|  |  |  |  |  | R:AGTGAAACTTTAATCAGTTG | 38 |  |  |  |  |
| Pdo 5 | *P. domesticus* | Griffith et al. (1999) | Y15126 | (TG)_21_ | F:GATGTTGCAGTGACCTCTCTT | 51 | 6-FAM | A | 210 - 266 | 200 - 266 |
|  |  |  |  |  | R:GCTGTGTTAATGCTAAAATGG | 53 |  |  |  |  |
| Pdo 10 | *P. domesticus* | Dawson et al. (2012) | FM865833 | (TG)_XX_ | F: ATGGTGAATCCCAGAAAC | 50 | 6-FAM | A | 112 - 145 | 80 - 145 |
|  |  |  |  |  | R:ATGGAGTTTGGGGAATGG | 49 |  |  |  |  |
| Pdo 16 | *P. domesticus* | Dawson et al. (2008, 2012) | AM158995.1  AM167523 | (CA)_14 or_ (CA)_28_ | F: GCAAATGACAAGACCAAAGCTTCA | 57 | Atto 565 | A | 170 - 196 | 148 - 195 |
|  |  |  |  |  | R:GGGAAGGAGGGCACAGGAGT | 57 |  |  |  |  |
| Pdo 17 | *P. domesticus* | Dawson et al. (2012) | AM158996 | (CA)_15_ GA (CA)_3_ GACG (CA)_2_ G (CA)_5_ TA (CA)_8_ | F:TGCCGTGAGGGATGTAAGTGA | 55 | HEX | B | 194 - 250 | 187 - 243 |
|  |  |  |  |  | R:CAGGCGACTTCAGCTTCTGC | 55 |  |  |  |  |
| Pdo19 | *P. domesticus* | Dawson et al. (2012) | AM158998 | (G)_4_ (GA)_15_ AA (GA)_3_ AAGAA AA | F:TCAGAGAGGGCAGAAGGGATTTC | 58 | HEX | C | 175 - 188 | 165 - 187 |
|  |  |  |  |  | R:GCACCGCAGGAGAGCACTTT | 57 |  |  |  |  |
| Pdo 22 | *P. domesticus* | Dawson et al. (2012) | AM159001 | (CA)_10_ (TA)_4_ | F:CATGGGCACAAGAAATGTGA | 52 | 6-FAM | C | 105 - 130 | 87 - 130 |
|  |  |  |  |  | R:TCAAGAAGAAAATGGGTAATATACTGG | 54 |  |  |  |  |
| Pdo 27 | *P. domesticus* | Dawson et al. (2012) | - | (CA)_11_ CT (CA)_4_ (CT)_2_ CA | F:TGGCAAGGAAGGAGGAATCG | 56 | 6-FAM | B | 230 - 260 | 222 - 273 |
|  |  |  |  |  | R:AGCAATATAAGGCCCAGGTGCTC | 58 |  |  |  |  |
| Pdo 30 | *P. domesticus* | Dawson et al. (2012) | AM159009 | (CA)_10_ (A)_5_ GC (CA)_3_ (A)_5_ | F: CCTGCATGCAAGATTTAACACA | 53 | Atto 550 | B | 184 - 206 | 168 - 202 |
|  |  |  |  |  | R: ACGGTCCATGTTGCCTAGC | 52 |  |  |  |  |
| Pdo 36 | *P. domesticus* | Dawson et al. (2012) | AM159015 | (GT)_19_ | F: GCATTCAAAAATGGCAAGAGGA | 56 | Atto 565 | B | 183 - 221 | 178 - 244 |
|  |  |  |  |  | R: GAGGCTACCCCTTTCCTGAACA | 56 |  |  |  |  |
| Pdo 44 | *P. domesticus* | Dawson et al. (2012) | AM159021 | (CA)_21_&(A ⁄ C)_37_ | F:ACAGCTGTCTAAAACACACACAC | 57 | Atto 565 | C | 215 - 248 | 199 - 253 |
|  |  |  |  |  | R:CTTCAGATGACTGGGGATTC | 56 |  |  |  |  |
| Pdo 47 | *P. domesticus* | Dawson et al. (2012) | AM159022 | (CA)21 | F: TGCACAGACTCCTGTACCTGTCA | 56 | 6-FAM | C | 165 - 197 | 149 - 267 |
|  |  |  |  |  | R: GCAAACACTCGACAGTGTGACC | 56 |  |  |  |  |

**Table S3: Observed and expected heterozygosity, departure from Hardy-Weinberg equilibrium (HWE) and linkage disequilibrium (LD) for each locus and population**; Bonferroni corrected p-value for HWE = 0.05/12 = 0.0042; Bonferroni corrected p-value for LD = 0.05/66 = 0.00076; departure from equilibrium is indicated in **bold**.

| **Kathmandu; Nepal (N = 21)** | | | | |
| --- | --- | --- | --- | --- |
| **Locus** | **Hardy-Weinberg-Equilibrium** | | | **Linkage Disequilibrium** |
|  | **Obs.Het.** | **Exp.Het.** | **P-value** |  |
| Pdo1 | 0.762 | 0.698 | 0.265 | none |
| Pdo3 | 0.700 | 0.795 | 0.310 |  |
| Pdo5 | 0.833 | 0.759 | 0.895 |  |
| Pdo10 | 0.857 | 0.836 | 0.875 |  |
| Pdo16 | 0.850 | 0.803 | 0.350 |  |
| Pdo17 | 0.619 | 0.501 | 0.358 |  |
| Pdo19 | 0.524 | 0.613 | 0.181 |  |
| Pdo22 | 0.889 | 0.800 | 0.886 |  |
| Pdo30 | 0.524 | 0.841 | **0.000** |  |
| Pdo36 | 0.400 | 0.705 | **0.001** |  |
| Pdo44 | 0.750 | 0.736 | 0.851 |  |
| Pdo47 | 0.789 | 0.795 | 0.563 |  |

| **Saxony, East Germany (N = 17)** | | | | |
| --- | --- | --- | --- | --- |
| **Locus** | **Hardy-Weinberg-Equilibrium** | | | **Linkage Disequilibrium** |
|  | **Obs.Het.** | **Exp.Het.** | **P-value** |  |
| Pdo1 | 0.875 | 0.861 | 0.911 | none |
| Pdo3 | 0.765 | 0.922 | 0.148 |  |
| Pdo5 | 0.765 | 0.840 | 0.037 |  |
| Pdo10 | 0.941 | 0.902 | 0.556 |  |
| Pdo16 | 0.636 | 0.892 | 0.091 |  |
| Pdo17 | 0.813 | 0.839 | 0.461 |  |
| Pdo19 | 0.882 | 0.626 | 0.111 |  |
| Pdo22 | 0.706 | 0.840 | 0.073 |  |
| Pdo30 | 0.688 | 0.726 | 0.503 |  |
| Pdo36 | 0.813 | 0.829 | 0.419 |  |
| Pdo44 | 0.941 | 0.909 | 0.861 |  |
| Pdo47 | 0.706 | 0.825 | 0.083 |  |

| **Landes, France (N = 12)** | | | | |
| --- | --- | --- | --- | --- |
| **Locus** | **Hardy-Weinberg-Equilibrium** | | | **Linkage Disequilibrium** |
|  | **Obs.Het.** | **Exp.Het.** | **P-value** |  |
| Pdo1 | 0.727 | 0.827 | 0.193 | Pdo3; Pdo5 |
| Pdo3 | 0.875 | 0.908 | 0.714 |  |
| Pdo5 | 1.000 | 0.915 | 1.000 |  |
| Pdo10 | 0.909 | 0.857 | 0.931 |  |
| Pdo16 | 0.727 | 0.918 | 0.007 |  |
| Pdo17 | 0.417 | 0.790 | **<0.001** |  |
| Pdo19 | 0.917 | 0.703 | 0.071 |  |
| Pdo22 | 0.750 | 0.841 | 0.157 |  |
| Pdo30 | 0.750 | 0.703 | 0.494 |  |
| Pdo36 | 0.833 | 0.859 | 0.893 |  |
| Pdo44 | 0.909 | 0.861 | 0.968 |  |
| Pdo47 | 0.750 | 0.888 | 0.037 |  |

| **Kefalonia, Greece (N = 4)** | | | | |
| --- | --- | --- | --- | --- |
| **Locus** | **Hardy-Weinberg-Equilibrium** | | | **Linkage Disequilibrium** |
|  | **Obs.Het.** | **Exp.Het.** | **P-value** |  |
| Pdo1 | 0.500 | 0.750 | 0.301 | none |
| Pdo3 | 0.750 | 0.893 | 0.353 |  |
| Pdo5 | 0.250 | 0.250 | 1.000 |  |
| Pdo10 | 0.500 | 0.750 | 0.084 |  |
| Pdo16 | 1.000 | 0.857 | 1.000 |  |
| Pdo17 | 0.750 | 0.929 | 0.295 |  |
| Pdo19 | 0.667 | 0.600 | 1.000 |  |
| Pdo22 | 1.000 | 0.867 | 1.000 |  |
| Pdo30 | 0.500 | 0.786 | 0.077 |  |
| Pdo36 | 0.500 | 0.929 | 0.029 |  |
| Pdo44 | 0.667 | 0.933 | 0.202 |  |
| Pdo47 | 0.667 | 0.867 | 0.484 |  |

| **Mŭgla, Turkey (N = 31)** | | | | |
| --- | --- | --- | --- | --- |
| **Locus** | **Hardy-Weinberg-Equilibrium** | | | **Linkage Disequilibrium** |
|  | **Obs.Het.** | **Exp.Het** | **P-value** |  |
| Pdo1 | 0.710 | 0.883 | **0.000** | none |
| Pdo3 | 0.767 | 0.903 | 0.043 |  |
| Pdo5 | 0.613 | 0.832 | **0.000** |  |
| Pdo10 | 0.774 | 0.919 | 0.064 |  |
| Pdo16 | 0.839 | 0.882 | 0.225 |  |
| Pdo17 | 0.871 | 0.921 | 0.705 |  |
| Pdo19 | 0.645 | 0.672 | 0.024 |  |
| Pdo22 | 0.710 | 0.826 | 0.113 |  |
| Pdo30 | 0.516 | 0.702 | 0.060 |  |
| Pdo36 | 0.871 | 0.887 | 0.130 |  |
| Pdo44 | 0.750 | 0.911 | 0.099 |  |
| Pdo47 | 0.903 | 0.916 | **0.000** |  |

| **Sevilla, Spain (*P.domesticus*) (N = 24)** | | | | |
| --- | --- | --- | --- | --- |
| **Locus** | **Hardy-Weinberg-Equilibrium** | | | **Linkage Disequilibrium** |
|  | **Obs.Het.** | **Exp.Het.** | **P-value** |  |
| Pdo1 | 0.792 | 0.911 | 0.539 | none |
| Pdo3 | 0.958 | 0.921 | 0.571 |  |
| Pdo5 | 0.833 | 0.887 | 0.833 |  |
| Pdo10 | 0.875 | 0.842 | 0.693 |  |
| Pdo16 | 0.875 | 0.892 | 0.115 |  |
| Pdo17 | 0.625 | 0.883 | **0.000** |  |
| Pdo19 | 0.542 | 0.630 | 0.660 |  |
| Pdo22 | 0.792 | 0.838 | 0.742 |  |
| Pdo30 | 0.875 | 0.819 | 0.187 |  |
| Pdo36 | 0.750 | 0.893 | 0.209 |  |
| Pdo44 | 0.792 | 0.885 | 0.119 |  |
| Pdo47 | 0.833 | 0.898 | 0.329 |  |

| **Sudan (N = 4)** | | | | |
| --- | --- | --- | --- | --- |
| **Locus** | **Hardy-Weinberg-Equilibrium** | | | **Linkage Disequilibrium** |
|  | **Obs.Het.** | **Exp.Het.** | **P-value** |  |
| Pdo1 | 0.750 | 0.857 | 0.629 | none |
| Pdo3 | 1.000 | 0.893 | 1.000 |  |
| Pdo5 | 0.500 | 0.893 | 0.144 |  |
| Pdo10 | 0.250 | 0.821 | 0.026 |  |
| Pdo16 | 0.750 | 0.929 | 0.271 |  |
| Pdo17 | 0.500 | 0.786 | 0.288 |  |
| Pdo19 | 0.000 | 0.429 | 0.137 |  |
| Pdo22 | 0.750 | 0.786 | 0.783 |  |
| Pdo30 | 0.250 | 0.750 | 0.137 |  |
| Pdo36 | 0.500 | 0.821 | 0.284 |  |
| Pdo44 | 0.500 | 0.464 | 1.000 |  |
| Pdo47 | 0.500 | 0.929 | 0.033 |  |

| **CentralAsia (*P. domesticus*) (N = 4)** | | | | |
| --- | --- | --- | --- | --- |
| **Locus** | **Hardy-Weinberg-Equilibrium** | | | **Linkage Disequilibrium** |
|  | **Obs.Het.** | **Exp.Het.** | **P-value** |  |
| Pdo1 | 0.750 | 0.929 | 0.284 | none |
| Pdo3 | 0.667 | 0.933 | 0.242 |  |
| Pdo5 | 0.750 | 0.893 | 0.117 |  |
| Pdo10 | 0.750 | 0.857 | 0.621 |  |
| Pdo16 | 0.500 | 0.786 | 0.065 |  |
| Pdo17 | 0.500 | 0.643 | 0.464 |  |
| Pdo19 | 0.250 | 0.536 | 0.424 |  |
| Pdo22 | 1.000 | 0.679 | 0.323 |  |
| Pdo30 | 0.250 | 0.607 | 0.131 |  |
| Pdo36 | 0.750 | 0.893 | 0.497 |  |
| Pdo44 | 0.750 | 0.893 | 0.463 |  |
| Pdo47 | 1.000 | 0.857 | 0.661 |  |

| **Morocco (N = 4)** | | | | |
| --- | --- | --- | --- | --- |
| **Locus** | **Hardy-Weinberg-Equilibrium** | | | **Linkage Disequilibrium** |
|  | **Obs.Het.** | **Exp.Het.** | **P-value** |  |
| Pdo1 | 0.750 | 0.893 | 0.449 | none |
| Pdo3 | 0.750 | 0.929 | 0.273 |  |
| Pdo5 | 0.667 | 0.733 | 1.000 |  |
| Pdo10 | 0.750 | 0.643 | 1.000 |  |
| Pdo16 | 0.750 | 0.929 | 0.332 |  |
| Pdo17 | 1.000 | 0.933 | 1.000 |  |
| Pdo19 | 0.500 | 0.679 | 1.000 |  |
| Pdo22 | 0.667 | 0.933 | 0.187 |  |
| Pdo30 | 0.333 | 0.333 | 1.000 |  |
| Pdo36 | 0.667 | 0.733 | 1.000 |  |
| Pdo44 | 1.000 | 1.000 | 1.000 |  |
| Pdo47 | 0.750 | 0.929 | 0.391 |  |

| **Djelfa (*P.domesticus*) (N = 28)** | | | | |
| --- | --- | --- | --- | --- |
| **Locus** | **Hardy-Weinberg-Equilibrium** | | | **Linkage Disequilibrium** |
|  | **Obs.Het.** | **Exp.Het.** | **P-value** |  |
| Pdo1 | 0.893 | 0.914 | 0.918 | Pdo44; Pdo47 |
| Pdo3 | 0.929 | 0.910 | 0.160 |  |
| Pdo5 | 0.821 | 0.879 | 0.285 |  |
| Pdo10 | 0.714 | 0.829 | 0.280 |  |
| Pdo16 | 0.852 | 0.897 | 0.606 |  |
| Pdo17 | 0.857 | 0.859 | 0.589 |  |
| Pdo19 | 0.429 | 0.592 | 0.022 |  |
| Pdo22 | 0.750 | 0.769 | 0.147 |  |
| Pdo30 | 0.679 | 0.742 | 0.061 |  |
| Pdo36 | 0.964 | 0.919 | 0.734 |  |
| Pdo44 | 0.630 | 0.910 | **0.000** |  |
| Pdo47 | 0.643 | 0.916 | **0.000** |  |

| **Djelfa (hybrids) (N = 16)** | | | | |
| --- | --- | --- | --- | --- |
| **Locus** | **Hardy-Weinberg-Equilibrium** | | | **Linkage Disequilibrium** |
|  | **Obs.Het.** | **Exp.Het.** | **P-value** |  |
| Pdo1 | 0.813 | 0.889 | 0.367 | none |
| Pdo3 | 0.938 | 0.861 | 0.991 |  |
| Pdo5 | 0.938 | 0.857 | 0.970 |  |
| Pdo10 | 0.750 | 0.847 | 0.259 |  |
| Pdo16 | 0.938 | 0.915 | 0.098 |  |
| Pdo17 | 0.813 | 0.863 | 0.159 |  |
| Pdo19 | 0.813 | 0.706 | 0.531 |  |
| Pdo22 | 0.938 | 0.827 | 0.472 |  |
| Pdo30 | 0.625 | 0.808 | **0.000** |  |
| Pdo36 | 0.875 | 0.899 | 0.036 |  |
| Pdo44 | 0.813 | 0.917 | 0.174 |  |
| Pdo47 | 1.000 | 0.919 | 0.841 |  |

| **Hassi-El Euch (*P.domesticus*) (N = 10)** | | | | |
| --- | --- | --- | --- | --- |
| **Locus** | **Hardy-Weinberg-Equilibrium** | | | **Linkage Disequilibrium** |
|  | **Obs.Het.** | **Exp.Het.** | **P-value** |  |
| Pdo1 | 0.778 | 0.837 | 0.668 | none |
| Pdo3 | 0.900 | 0.942 | 0.489 |  |
| Pdo5 | 0.800 | 0.842 | 0.430 |  |
| Pdo10 | 0.800 | 0.889 | 0.563 |  |
| Pdo16 | 1.000 | 0.926 | 0.549 |  |
| Pdo17 | 0.900 | 0.895 | 0.950 |  |
| Pdo19 | 0.778 | 0.758 | 1.000 |  |
| Pdo22 | 0.700 | 0.837 | 0.416 |  |
| Pdo30 | 0.600 | 0.784 | 0.136 |  |
| Pdo36 | 1.000 | 0.889 | 1.000 |  |
| Pdo44 | 0.200 | 0.858 | **0.000** |  |
| Pdo47 | 0.800 | 0.874 | 0.371 |  |

| **Hassi-El Euch (hybrids) (N = 9)** | | | | |
| --- | --- | --- | --- | --- |
| **Locus** | **Hardy-Weinberg-Equilibrium** | | | **Linkage Disequilibrium** |
|  | **Obs.Het.** | **Exp.Het.** | **P-value** |  |
| Pdo1 | 1.000 | 0.915 | 0.361 | none |
| Pdo3 | 0.778 | 0.882 | 0.242 |  |
| Pdo5 | 1.000 | 0.941 | 1.000 |  |
| Pdo10 | 0.667 | 0.745 | 0.643 |  |
| Pdo16 | 0.667 | 0.869 | 0.107 |  |
| Pdo17 | 0.778 | 0.863 | 0.674 |  |
| Pdo19 | 0.444 | 0.523 | 0.439 |  |
| Pdo22 | 0.778 | 0.869 | 0.254 |  |
| Pdo30 | 0.778 | 0.810 | 0.265 |  |
| Pdo36 | 0.667 | 0.915 | 0.038 |  |
| Pdo44 | 0.889 | 0.935 | 0.212 |  |
| Pdo47 | 0.889 | 0.817 | 0.763 |  |

| **Fuerteventura, Spain (N = 19)** | | | | |
| --- | --- | --- | --- | --- |
| **Locus** | **Hardy-Weinberg-Equilibrium** | | | **Linkage Disequilibrium** |
|  | **Obs.Het.** | **Exp.Het.** | **P-value** |  |
| Pdo1 | 0.895 | 0.844 | 0.112 | none |
| Pdo3 | 0.737 | 0.876 | 0.363 |  |
| Pdo5 | 0.842 | 0.868 | 0.867 |  |
| Pdo10 | 0.211 | 0.374 | 0.031 |  |
| Pdo16 | 0.947 | 0.868 | 0.640 |  |
| Pdo17 | 0.579 | 0.553 | 1.000 |  |
| Pdo19 | 0.316 | 0.428 | 0.094 |  |
| Pdo22 | 0.526 | 0.708 | 0.000 |  |
| Pdo30 | 0.611 | 0.790 | 0.122 |  |
| Pdo36 | 0.722 | 0.676 | 0.930 |  |
| Pdo44 | 0.789 | 0.885 | 0.068 |  |
| Pdo47 | 0.842 | 0.802 | 0.377 |  |

| **Giza, Egypt (N = 6)** | | | | |
| --- | --- | --- | --- | --- |
| **Locus** | **Hardy-Weinberg-Equilibrium** | | | **Linkage Disequilibrium** |
|  | **Obs.Het.** | **Exp.Het.** | **P-value** |  |
| Pdo1 | 1.000 | 0.894 | 1.000 | none |
| Pdo3 | 1.000 | 0.909 | 1.000 |  |
| Pdo5 | 1.000 | 0.894 | 0.772 |  |
| Pdo10 | 0.833 | 0.530 | 0.400 |  |
| Pdo16 | 0.833 | 0.894 | 0.753 |  |
| Pdo17 | 0.833 | 0.970 | 0.197 |  |
| Pdo19 | 0.667 | 0.530 | 1.000 |  |
| Pdo22 | 0.833 | 0.924 | 0.470 |  |
| Pdo30 | 0.667 | 0.833 | 0.523 |  |
| Pdo36 | 0.667 | 0.773 | 0.274 |  |
| Pdo44 | 1.000 | 0.955 | 1.000 |  |
| Pdo47 | 0.667 | 0.803 | 0.762 |  |

| **Lybia (N = 4)** | | | | |
| --- | --- | --- | --- | --- |
| **Locus** | **Hardy-Weinberg-Equilibrium** | | | **Linkage Disequilibrium** |
|  | **Obs.Het.** | **Exp.Het.** | **P-value** |  |
| Pdo1 | 0.667 | 0.933 | 0.208 | none |
| Pdo3 | 1.000 | 1.000 | 1.000 |  |
| Pdo5 | 0.750 | 0.607 | 1.000 |  |
| Pdo10 | 0.500 | 0.750 | 0.655 |  |
| Pdo16 | monomorphic: | | |  |
| Pdo17 | 1.000 | 0.867 | 1.000 |  |
| Pdo19 | 0.667 | 0.533 | 1.000 |  |
| Pdo22 | 1.000 | 0.929 | 1.000 |  |
| Pdo30 | 0.667 | 0.733 | 1.000 |  |
| Pdo36 | monomorphic: | | |  |
| Pdo44 | 0.500 | 0.786 | 0.317 |  |
| Pdo47 | 1.000 | 0.893 | 1.000 |  |

| **Sevilla, Spain (*P.hispaniolensis*) (N = 24)** | | | | |
| --- | --- | --- | --- | --- |
| **Locus** | **Hardy-Weinberg-Equilibrium** | | | **Linkage Disequilibrium** |
|  | **Obs.Het.** | **Exp.Het.** | **P-value** |  |
| Pdo1 | 0.783 | 0.895 | 0.817 | none |
| Pdo3 | 0.913 | 0.906 | 0.868 |  |
| Pdo5 | 0.826 | 0.895 | **0.000** |  |
| Pdo10 | 0.478 | 0.558 | 0.735 |  |
| Pdo16 | 0.913 | 0.885 | 0.613 |  |
| Pdo17 | 0.870 | 0.834 | 1.000 |  |
| Pdo19 | 0.478 | 0.519 | 0.496 |  |
| Pdo22 | 0.783 | 0.853 | 0.689 |  |
| Pdo30 | 0.783 | 0.716 | 0.791 |  |
| Pdo36 | 0.870 | 0.826 | 0.614 |  |
| Pdo44 | 0.870 | 0.931 | 0.110 |  |
| Pdo47 | 0.870 | 0.909 | 0.060 |  |

| **Centra Asia (*P. hispaniolensis*) (N = 3)** | | | | |
| --- | --- | --- | --- | --- |
| **Locus** | **Hardy-Weinberg-Equilibrium** | | | **Linkage Disequilibrium** |
|  | **Obs.Het.** | **Exp.Het.** | **P-value** |  |
| Pdo1 | 1.000 | 0.933 | 1.000 | none |
| Pdo3 | 1.000 | 1.000 | 1.000 |  |
| Pdo5 | 1.000 | 0.867 | 1.000 |  |
| Pdo10 | 1.000 | 0.600 | 0.394 |  |
| Pdo16 | 0.667 | 0.867 | 0.474 |  |
| Pdo17 | 1.000 | 0.867 | 1.000 |  |
| Pdo19 | 0.667 | 0.800 | 0.587 |  |
| Pdo22 | 0.667 | 0.600 | 1.000 |  |
| Pdo30 | 0.667 | 0.600 | 1.000 |  |
| Pdo36 | 0.667 | 0.933 | 0.198 |  |
| Pdo44 | 1.000 | 0.933 | 1.000 |  |
| Pdo47 | 1.000 | 0.867 | 1.000 |  |

| **Hassi-El Euch (*P. hispaniolensis*) (N = 25)** | | | | |
| --- | --- | --- | --- | --- |
| **Locus** | **Hardy-Weinberg-Equilibrium** | | | **Linkage Disequilibrium** |
|  | **Obs.Het.** | **Exp.Het.** | **P-value** |  |
| Pdo1 | 0.840 | 0.901 | 0.252 | none |
| Pdo3 | 0.920 | 0.886 | 0.602 |  |
| Pdo5 | 0.800 | 0.860 | 0.073 |  |
| Pdo10 | 0.760 | 0.845 | 0.132 |  |
| Pdo16 | 0.875 | 0.880 | 0.901 |  |
| Pdo17 | 0.800 | 0.857 | 0.201 |  |
| Pdo19 | 0.640 | 0.554 | 0.598 |  |
| Pdo22 | 0.840 | 0.850 | 0.938 |  |
| Pdo30 | 0.800 | 0.778 | 0.673 |  |
| Pdo36 | 0.680 | 0.867 | 0.144 |  |
| Pdo44 | 0.750 | 0.895 | 0.149 |  |
| Pdo47 | 0.920 | 0.874 | 0.111 |  |

| **Sicily East, Maletto (Italy) (N = 11)** | | | | |
| --- | --- | --- | --- | --- |
| **Locus** | **Hardy-Weinberg-Equilibrium** | | | **Linkage Disequilibrium** |
|  | **Obs.Het.** | **Exp.Het.** | **P-value** |  |
| Pdo1 | 0.900 | 0.916 | 0.793 | Pdo16; Pdo36 |
| Pdo3 | 1.000 | 0.944 | 1.000 |  |
| Pdo5 | 0.636 | 0.905 | 0.081 |  |
| Pdo10 | 0.909 | 0.727 | 0.953 |  |
| Pdo16 | 0.727 | 0.840 | 0.429 |  |
| Pdo17 | 0.909 | 0.827 | 0.927 |  |
| Pdo19 | 0.182 | 0.255 | 0.145 |  |
| Pdo22 | 0.818 | 0.870 | 0.440 |  |
| Pdo30 | 0.909 | 0.827 | 0.293 |  |
| Pdo36 | 0.909 | 0.801 | 0.619 |  |
| Pdo44 | 1.000 | 0.926 | 1.000 |  |
| Pdo47 | 1.000 | 0.926 | 0.698 |  |

| **Sicily West, Fraginesi (Italy) (N = 10)** | | | | |
| --- | --- | --- | --- | --- |
| **Locus** | **Hardy-Weinberg-Equilibrium** | | | **Linkage Disequilibrium** |
|  | **Obs.Het.** | **Exp.Het.** | **P-value** |  |
| Pdo1 | 0.900 | 0.926 | 0.855 | none |
| Pdo3 | 0.889 | 0.941 | 0.357 |  |
| Pdo5 | 1.000 | 0.928 | 1.000 |  |
| Pdo10 | 0.667 | 0.758 | 0.146 |  |
| Pdo16 | 0.889 | 0.902 | 0.890 |  |
| Pdo17 | 0.400 | 0.647 | **0.000** |  |
| Pdo19 | 0.400 | 0.442 | 1.000 |  |
| Pdo22 | 0.700 | 0.689 | 0.934 |  |
| Pdo30 | 0.700 | 0.653 | 0.745 |  |
| Pdo36 | 1.000 | 0.832 | 0.136 |  |
| Pdo44 | 0.900 | 0.926 | 0.762 |  |
| Pdo47 | 1.000 | 0.900 | 0.903 |  |

| **Lampedusa Island (Italy) (N = 14)** | | | | |
| --- | --- | --- | --- | --- |
| **Locus** | **Hardy-Weinberg-Equilibrium** | | | **Linkage Disequilibrium** |
|  | **Obs.Het.** | **Exp.Het.** | **P-value** |  |
| Pdo1 | 0.714 | 0.765 | 0.207 | none |
| Pdo3 | 0.857 | 0.836 | 0.901 |  |
| Pdo5 | 0.929 | 0.855 | 1.000 |  |
| Pdo10 | 0.714 | 0.783 | 0.710 |  |
| Pdo16 | 0.929 | 0.889 | 0.791 |  |
| Pdo17 | 0.643 | 0.807 | 0.180 |  |
| Pdo19 | 0.714 | 0.648 | 0.716 |  |
| Pdo22 | 0.857 | 0.775 | 0.529 |  |
| Pdo30 | 0.571 | 0.852 | 0.051 |  |
| Pdo36 | 0.643 | 0.698 | 0.193 |  |
| Pdo44 | 0.857 | 0.899 | 0.519 |  |
| Pdo47 | 0.643 | 0.630 | 0.465 |  |

| **Ustica Island (Italy) (N = 10)** | | | | |
| --- | --- | --- | --- | --- |
| **Locus** | **Hardy-Weinberg-Equilibrium** | | | **Linkage Disequilibrium** |
|  | **Obs.Het.** | **Exp.Het.** | **P-value** |  |
| Pdo1 | 0.889 | 0.869 | 0.971 | none |
| Pdo3 | 0.900 | 0.911 | 0.878 |  |
| Pdo5 | 0.600 | 0.884 | **0.002** |  |
| Pdo10 | 0.900 | 0.795 | 0.992 |  |
| Pdo16 | 0.800 | 0.847 | 0.164 |  |
| Pdo17 | 0.889 | 0.843 | 0.983 |  |
| Pdo19 | 0.800 | 0.653 | 0.282 |  |
| Pdo22 | 0.600 | 0.747 | 0.260 |  |
| Pdo30 | 0.778 | 0.804 | 0.883 |  |
| Pdo36 | 0.889 | 0.856 | 0.971 |  |
| Pdo44 | 0.900 | 0.895 | 0.710 |  |
| Pdo47 | 1.000 | 0.837 | 0.184 |  |

| **Lipari Island (Italy) (N = 5)** | | | | |
| --- | --- | --- | --- | --- |
| **Locus** | **Hardy-Weinberg-Equilibrium** | | | **Linkage Disequilibrium** |
|  | **Obs.Het.** | **Exp.Het.** | **P-value** |  |
| Pdo1 | 0.800 | 0.933 | 0.261 | none |
| Pdo3 | 0.600 | 0.800 | 0.506 |  |
| Pdo5 | 0.800 | 0.911 | 0.530 |  |
| Pdo10 | 0.400 | 0.733 | 0.120 |  |
| Pdo16 | 1.000 | 0.933 | 1.000 |  |
| Pdo17 | 1.000 | 0.956 | 1.000 |  |
| Pdo19 | 1.000 | 0.689 | 0.430 |  |
| Pdo22 | 0.600 | 0.889 | 0.131 |  |
| Pdo30 | 0.800 | 0.844 | 0.875 |  |
| Pdo36 | 1.000 | 0.822 | 0.937 |  |
| Pdo44 | 0.800 | 0.800 | 0.628 |  |
| Pdo47 | 1.000 | 0.911 | 1.000 |  |

| **La Chiappa, Corsica (France) (N = 4)** | | | | |
| --- | --- | --- | --- | --- |
| **Locus** | **Hardy-Weinberg-Equilibrium** | | | **Linkage Disequilibrium** |
|  | **Obs.Het.** | **Exp.Het.** | **P-value** |  |
| Pdo1 | 1.000 | 0.857 | 1.000 | none |
| Pdo3 | 0.750 | 0.821 | 0.786 |  |
| Pdo5 | 1.000 | 0.893 | 1.000 |  |
| Pdo10 | 1.000 | 0.929 | 1.000 |  |
| Pdo16 | 0.750 | 0.929 | 0.347 |  |
| Pdo17 | 1.000 | 0.893 | 1.000 |  |
| Pdo19 | 0.250 | 0.250 | 1.000 |  |
| Pdo22 | 0.500 | 0.464 | 1.000 |  |
| Pdo30 | 0.750 | 0.786 | 1.000 |  |
| Pdo36 | 1.000 | 0.679 | 0.305 |  |
| Pdo44 | 0.500 | 0.643 | 0.454 |  |
| Pdo47 | 1.000 | 0.964 | 1.000 |  |

| **Pantelleria Island (Italy) (N = 4)** | | | | |
| --- | --- | --- | --- | --- |
| **Locus** | **Hardy-Weinberg-Equilibrium** | | | **Linkage Disequilibrium** |
|  | **Obs.Het.** | **Exp.Het.** | **P-value** |  |
| Pdo1 | 0.500 | 0.833 | 0.316 | none |
| Pdo3 | 0.750 | 0.929 | 0.246 |  |
| Pdo5 | 1.000 | 0.964 | 1.000 |  |
| Pdo10 | 0.750 | 0.857 | 0.649 |  |
| Pdo16 | 0.500 | 0.857 | 0.093 |  |
| Pdo17 | 1.000 | 1.000 | 1.000 |  |
| Pdo19 | 0.750 | 0.607 | 1.000 |  |
| Pdo22 | 0.750 | 0.750 | 0.303 |  |
| Pdo30 | 0.500 | 0.500 | 1.000 |  |
| Pdo36 | 0.500 | 0.500 | 1.000 |  |
| Pdo44 | 1.000 | 0.893 | 1.000 |  |
| Pdo47 | 0.750 | 0.929 | 0.341 |  |

Table S4: Simulated data: 17 house sparrow (*P. domesticus*) samples from Germany and 22 Spanish sparrow (*P. hispaniolensis*) samples from Fuerteventura were chosen as pure parental genotypes. Using this data, 20 genotypes of each hybrid class (F1, F2, and the two backcrosses) were modelled in hybridlab and analyzed with structure; for a threshold of assignment probability 0.9 only a minor percentage of the simulated parental populations were misassigned (q < 0.9; misassignment 5 - 6%).

|  | ***P.domesticus*** | ***P. hispaniolensis*** | **F1** | **F2** | **Backcross *DOM*** | **Backcross *HIS*** |
| --- | --- | --- | --- | --- | --- | --- |
| **structure (microsatellites)** |  |  |  |  |  |  |
| Average *Q* score | 0.951 | 0.952 | 0.484 | 0.548 | 0.860 | 0.862 |
| SD | 0.004 | 0.003 | 0.270 | 0.292 | 0.133 | 0.138 |
| MIN | 0.792 | 0.880 | 0.194 | 0.031 | 0.407 | 0.435 |
| MAX | 0.984 | 0.982 | 0.806 | 0.969 | 0.968 | 0.964 |
| Misassignment (if q < 0.9) | 6% | 5% | 25% | 45% | 55% | 55% |
